# Curcumin activates a ROS/KEAP1/NRF2/*miR-34a/b/c* cascade to suppress colorectal cancer metastasis

**DOI:** 10.1038/s41418-023-01178-1

**Published:** 2023-05-20

**Authors:** Chunfeng Liu, Matjaz Rokavec, Zekai Huang, Heiko Hermeking

**Affiliations:** 1 Experimental and Molecular Pathology, Institute of Pathology, Ludwig-Maximilians-Universität, Thalkirchner Strasse 36, 80337 Munich, Germany; 2 German Cancer Consortium (DKTK), Partner Site Munich, 80336 Munich, Germany; 3 German Cancer Research Center (DKFZ), 69120 Heidelberg, Germany

**Keywords:** Tumour-suppressor proteins, Translational research

## Abstract

Curcumin, a natural phytochemical isolated from tumeric roots, represents a candidate for prevention and therapy of colorectal cancer/CRC. However, the exact mechanism of action and the downstream mediators of curcumin’s tumor suppressive effects have remained largely unknown. Here we used a genetic approach to determine the role of the p53/miR-34 pathway as mediator of the effects of curcumin. Three isogenic CRC cell lines rendered deficient for the *p53*, *miR-34a* and/or *miR-34b/c* genes were exposed to curcumin and subjected to cell biological analyses. siRNA-mediated inhibition and ectopic expression of NRF2, as well as Western blot, qPCR and qChIP analyses of its target genes were performed. CRC cells were i.v. injected into NOD/SCID mice and lung-metastases formation was determined by longitudinal, non-invasive imaging. In CRC cells curcumin induced apoptosis and senescence, and suppressed migration and invasion in a p53-independent manner. Curcumin activated the KEAP1/NRF2/ARE pathway by inducing ROS. Notably, curcumin induced *miR-34a* and *miR-34b/c* expression in a ROS/NRF2-dependent and p53-independent manner. NRF2 directly induced *miR-34a* and *miR-34b/c* via occupying multiple ARE motifs in their promoter regions. Curcumin reverted repression of *miR-34a* and *miR-34b/c* induced by IL6 and hypoxia. Deletion of *miR-34a* and *miR-34b/c* significantly reduced curcumin-induced apoptosis and senescence, and prevented the inhibition of migration and invasion by curcumin or ectopic NRF2. In CRC cells curcumin induced MET and prevented the formation of lung-metastases in mice in a miR-34a-dependent manner. In addition, we found that curcumin may enhance the therapeutic effects of 5-FU on CRC cells deficient for *p53* and *miR-34a/b/c*. Activation of the KEAP1/NRF2/miR-34a/b/c axis mediates the tumor suppressive activity of curcumin and suggests a new approach for activating *miR-34* genes in tumors for therapeutic purposes.

## Introduction

Colorectal cancer (CRC) is the 2^nd^ most lethal cancer causing more than 900,000 deaths world-wide every year [1]. It has been estimated that over half of the Western population develops benign adenomatous polyps during its lifetime, and that 10% of these tumors proceed to malignant colorectal carcinomas [2]. Unfortunately, despite recent advances in currently available therapies, the 5-year survival rate of patients with metastatic CRC remains poor (<15%) [3]. Therefore, new approaches and compounds for improved prevention and therapy of CRC are clearly needed.

Curcumin is a polyphenol derived from the rhizome of the turmeric plant (*Curcuma longa*) and has been a popular food additive in Eastern cuisine. In addition, it has been used for centuries in traditional Chinese and ayurvedic medicine. Notably, curcumin has potential as a preventive and therapeutic agent for CRC, as it suppresses many hallmarks of cancer cells and exhibited promising effects in preclinical and clinical studies [4–6]. For example, the addition of daily oral curcumin to FOLFOX chemotherapy (folic acid/5-fluorouracil/oxaliplatin) significantly prolonged the progression-free survival and overall survival) of patients with metastatic CRC [7]. Moreover, curcumin showed improved erytrocyte sedimentation rate and C-reactive protein/CRP serum levels of in stage 3 CRC patients and improved their quality of life [8]. Daily oral curcumin given to patients with advanced colorectal cancer refractory to standard chemotherapy, resulted in stable disease in 5 of 15 individuals within 4 months of follow-up evaluation [9]. When curcumin was given in combination with mesalamine it resulted in remissions of patients with ulcerative colitis/UC [10, 11]. Furthermore, in familial adenomatous polyposis/FAP patients a combination of curcumin and quercetin reduced the number and size of ileal and rectal adenomas without appreciable toxicity [12]. As the clinical studies only included small numbers of patients, larger, targeted and prospective clinical trials are required to establish curcumin in clinical practice.

Curcumin was shown to affect the expression of non-coding RNAs in CRC cells [13]. Also miR-34a, a p53-inducible microRNA with tumor-suppressive capacities [14, 15], was induced by exposure to curcumin. However, it remained unclear whether this effect of curcumin was mediated by p53 activation. The *miR-34a* and *miR-34b/c* genes are down-regulated by p53 inactivation, epigenetic silencing or activation of HIF1A and STAT3, which directly suppress the *miR-34a* and *miR-34b/c* genes in the majority of CRCs [16–19]. Loss of *miR-34a* promotes colitis-associated colon cancer by activating the IL6-STAT3 pathway [20]. Furthermore, concomitant loss of *p53* and *miR-34a* enhances colorectal cancer formation in mouse model of sporadic CRC and is associated with poor survival of CRC patients [21]. In addition, deletion of *miR-34a* in the *Apc*^*Min/+*^ mouse model of inherited colon cancer enhances the number and size of adenomas and shortens the life span of mice [22]. Therefore, down-regulation of *miR-34a/b/c* in CRCs does not only commonly occur during intestinal carcinogenesis, but is also causally involved in CRC formation. Taken together, these observations suggest that *miR-34a* and *miR-34b/c* have tumor suppressive properties and would therefore represent attractive mediators of tumor prevention and/or suppression elicited by curcumin.

Here we show that curcumin induces the expression of *miR-34a* and *miR-34b/c* independent of p53 by activating the KEAP1/NRF2/ARE pathway via the generation of reactive oxygen species/ROS. Furthermore, we demonstrate that the *miR-34a/b/c* genes are important mediators of the pro-apoptotic, senescence-inducing and metastasis-inhibiting effects of curcumin. Importantly, these effects of curcumin are independent of p53 and dominant over signals from the micro-environment that repress miR-34 expression, such as hypoxia. Therefore, the identified mechanisms provide an opportunity to activate *miR-34a* and *miR-34b/c* genes in tumor cells that display inactivation/down-regulation of the p53/miR-34 pathway.

## Results

### *p53*-independent effects of curcumin on CRC cells

**Fig. 1:**
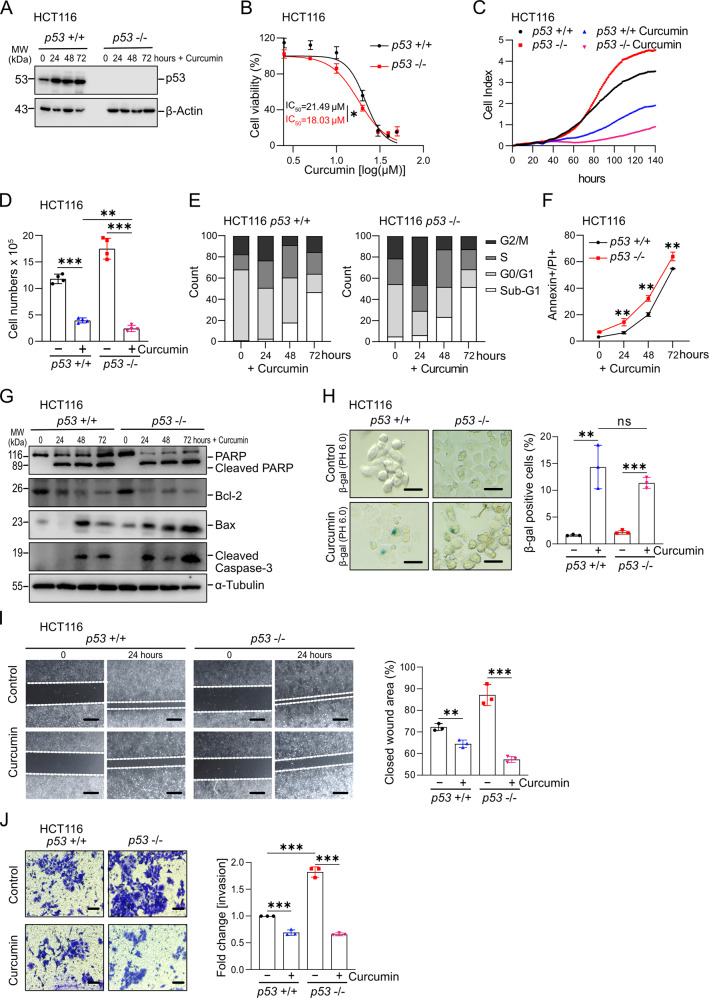
*p53*-independent effects of curcumin on CRC cells. **A** Detection of p53 protein by Western blot analysis after treatment with curcumin. β-Actin served as a loading control. **B** Cell viability of HCT116 cells exposed to different concentrations of curcumin for 48 hours was determined by MTT assays. **C** Impedance of HCT116 cells treated with curcumin. **D** Determination of cell number at the final time point of the experiment shown in (**C**). **E** Cell cycle analysis using propidium iodide (PI) staining. **F** Analysis of apoptosis in HCT116 cells treated with curcumin determined by Annexin V FITC and propidium iodide staining. **G** The level of cleaved PARP, Bcl-2, Bax, and cleaved caspase-3 was analyzed by Western blot analysis after being treated with curcumin for the indicated periods in HCT116 cells. α-Tubulin served as a loading control. **H** Detection of senescent cells after exposure to curcumin for 48 hours determined by pH 6 β-gal staining. Scale bars: 100 μm. **I** Wound healing assay of HCT116 cells treated with curcumin for 24 hours (left panel). Results represent the mean (%) of wound closure (right panel). **J** Determination of invasion in a modified Boyden-chamber assay. Relative invasion of HCT116 cells treated with curcumin for 48 hours. Scale bars: 100 μm. In panels (**B**), (**C**), (**F**), (**H**), (**I**), and (**J**) (n = 3), and (**D**) (n = 4) mean values ± SD are shown. *P <  0.05, **P  <  0.01, ***P <  0.001.

As shown previously [23], the amount of p53 protein increased after treatment of HCT116 cells with curcumin (Fig. 1A). In order to determine the relevance of p53 for the effects of curcumin on CRC cells, we treated HCT116 with wild-type *p53* and isogenic HCT116 cells with homozygous deletion of *p53* with increasing concentrations of curcumin for 48 hours. *P53*-proficient cells displayed an IC_50_ of 21.49 μM, whereas *p53*-deficient cells had an IC_50_ of 18.03 μM curcumin (Fig. 1B). Similar effects were observed in *p53*-proficient or *p53*-deficient RKO and SW48 CRC cell lines (Figs. S1A and 1D). A concentration of 15 μM curcumin, slightly below the IC_50_ value of *p53*-deficient cells, was chosen for the following experiments. Exposure to curcumin strongly reduced the proliferation of both *p53*-deficient and *p53*-proficient HCT116 cells as determined by impedance measurements (Fig. 1C) and cell number alterations were confirmed at the final time point (Fig. 1D). Similar *p53*-independent effects of curcumin were observed in the CRC cell lines RKO and SW48 (Figs. S1B, 1C, 1E, and 1F). Interestingly, the curcumin-induced decrease in viability and proliferation was more pronounced in *p53*-deficient cells. Therefore, these effects of curcumin on CRC cells are independent of p53, although p53 accumulates after treatment with curcumin.

Next, we analysed which processes may underlie the curcumin-mediated suppression of proliferation. Curcumin resulted in an increase of cells with sub-G_1_ DNA content, indicating increased apoptosis, irrespective of the *p53* status (Fig. 1E). *p53*-deficient cells displayed more G_2_/M-arrest 24 hours after curcumin treatment, whereas cells accumulated more in G_0_/G_1_ in WT *p53* cells. Annexin V/PI detection revealed a more pronounced increase in apoptosis in *p53*-deficient when compared to *p53*-proficient cells (Figs. 1F and S1G). These results were confirmed by detection of cleaved-PARP, cleaved Caspase-3, Bcl-2, and Bax proteins (Figs. 1G, S1H, and S1I): an increase in BAX and cleaved Caspase 3 was detected by 24 hours in *p53*-deficient cells, whereas *p53*-proficient cells showed a delayed induction of these proteins by 48 hours. In addition, curcumin induced senescence in HCT116 cells to a similar degree in *p53*-proficient and *p53*-deficient HCT116 cells as determined by detection of β-gal pH 6 (Fig. 1H). Finally, curcumin suppressed migration and invasion in *p53*-proficient and *p53*-deficient HCT116 cells, with the latter displaying increased basal levels of migration and invasion (Fig. 1I, J). Taken together, curcumin suppressed cell viability, proliferation, migration and invasion, whereas it induced apoptosis and senescence of CRC cells in a p53-independent manner.

### Curcumin activates NRF2 via ROS in CRC cells

**Fig. 2:**
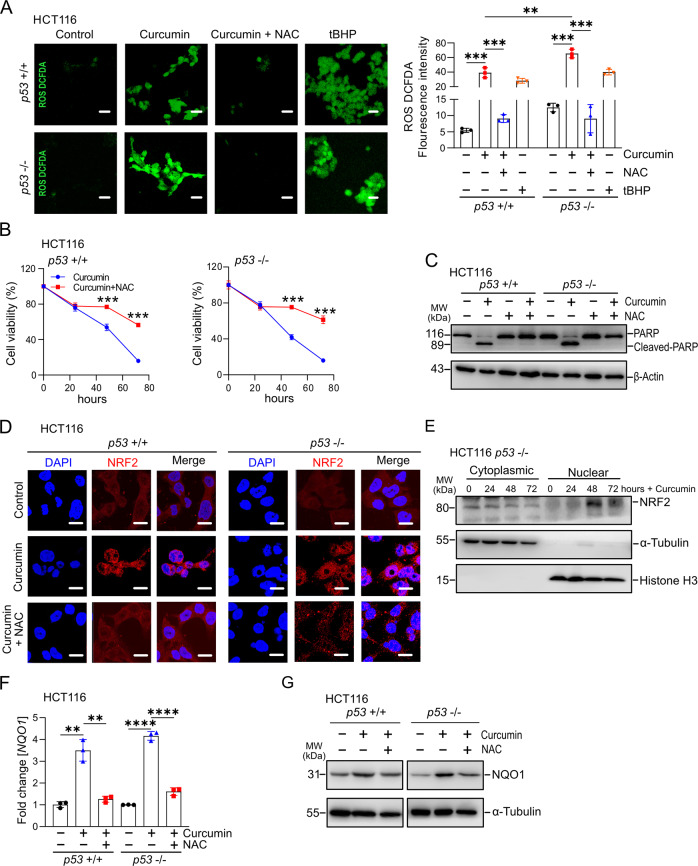
Curcumin activates NRF2 via inducing ROS in CRC cells. **A** Analysis of ROS formation in HCT116 cells treated as indicated, Left panel**:** representative IF pictures. Scale bar: 100 µM. Right panel: Quantification of fluorescence intensity. **B** Cell viability of HCT116 cells after treatment with curcumin and NAC for indicated periods was determined by MTT assays. **C** Apoptosis of HCT116 cells treated with curcumin and/or NAC for 48 h was determined by Western blot analysis of cleaved PARP protein levels. β-actin served as a loading control. **D** Immunofluorescence detection of NRF2 protein after treatment with DMSO, 15 μM curcumin, or 15 μM curcumin and 5 mM NAC for 48 hours. Nuclear DNA was detected by DAPI. Scale bar represents 20 µm. **E** Western blot analysis of NRF2 protein levels in cytoplasmic and nuclear cellular fractions after curcumin treatment. **F** qPCR analysis of *NQO1* expression in HCT116 cells after treatment with DMSO, 15 μM curcumin, or 15 μM curcumin with 5 mM NAC for 48 hours. **G** Western blot analysis of NQO1 protein levels after treatment with DMSO, 15 μM curcumin, or 15 μM curcumin with 5 mM NAC for 48 hours. In panels **A**, **B**, and **F** mean values ± SD are shown (n = 3). *P <  0.05, **P <  0.01, ***P <  0.001, ****P  <  0.0001.

Next, we aimed to determine the mechanism mediating the effects of curcumin on CRC cells. Although curcumin is mainly known for its anti-oxidative effects, it has also been shown to increase the production of reactive oxygen species/ROS [24]. Indeed, ROS levels of HCT116 cells were significantly increased when exposed to curcumin for 48 hours (Fig. 2A). The ROS-inducer tert-butyl hydroperoxide (tBHP) was used as a positive control. The effect of curcumin was suppressed by concomitant treatment with the antioxidant N-acetylcysteine (NAC) (Fig. 2A, left panel). Similar results were observed in RKO and SW48 cells (Figs. S2A and 2B, left panel). Interestingly, the levels of ROS were higher in *p53*-deficient HCT116 cells than in *p53*-proficient HCT116 cells, suggesting that p53 suppresses the production of ROS by curcumin (Fig. 2A, right panel). Similar results were obtained in RKO and SW48 cells (Figs. S2A and 2B, right panel). Notably, the negative effect of curcumin on cell viability was partially reversed by NAC in *p53*-proficient and *p53*-deficient HCT116 cells (Fig. 2B). Similar results were observed in RKO and SW48 cells (Figs. S2C and 2D). NAC completely suppressed cleavage of PARP and therefore apoptosis induced by curcumin (Fig. 2C). Similar results were observed in RKO and SW48 cells (Fig. S3B).

The KEAP1/NRF2-pathway is a central mediator of the cellular response to oxidative stress [25]. Upon exposure to ROS the transcription factor NRF2 is released from Keap1 and translocates from the cytoplasm to the nucleus to activate the transcription of its target genes [26]. Indeed, curcumin induced translocation of NRF2 to the nucleus in *p53*-proficient and *p53*-deficient HCT116 cells (Fig. 2D). Similar results were observed in RKO and SW48 cells (Fig. S3A). After exposure to curcumin, the NRF2 protein was increased in the nuclear fraction of HCT116 cells, whereas it decreased in the cytoplasmic fraction (Fig. 2E). *NRF2* mRNA expression was not affected by curcumin (Fig. S3C). *NQO1* is a conserved target gene of *NRF2* that is commonly used to monitor the activity of the NRF2 pathway [27]. Consistent with an activation of NRF2, expression of *NQO1* mRNA and protein was increased upon curcumin treatment (Figs. 2F, G). Similar results were observed in RKO and SW48 cells (Fig. S3B). Importantly, the induction of nuclear translocation and activation of NRF2 was largely reversed by the inhibition of ROS with NAC (Figs. 2D, F, G and S3A).

### Curcumin up-regulates *miR-34a* and *miR-34b/c* independent of p53

**Fig. 3:**
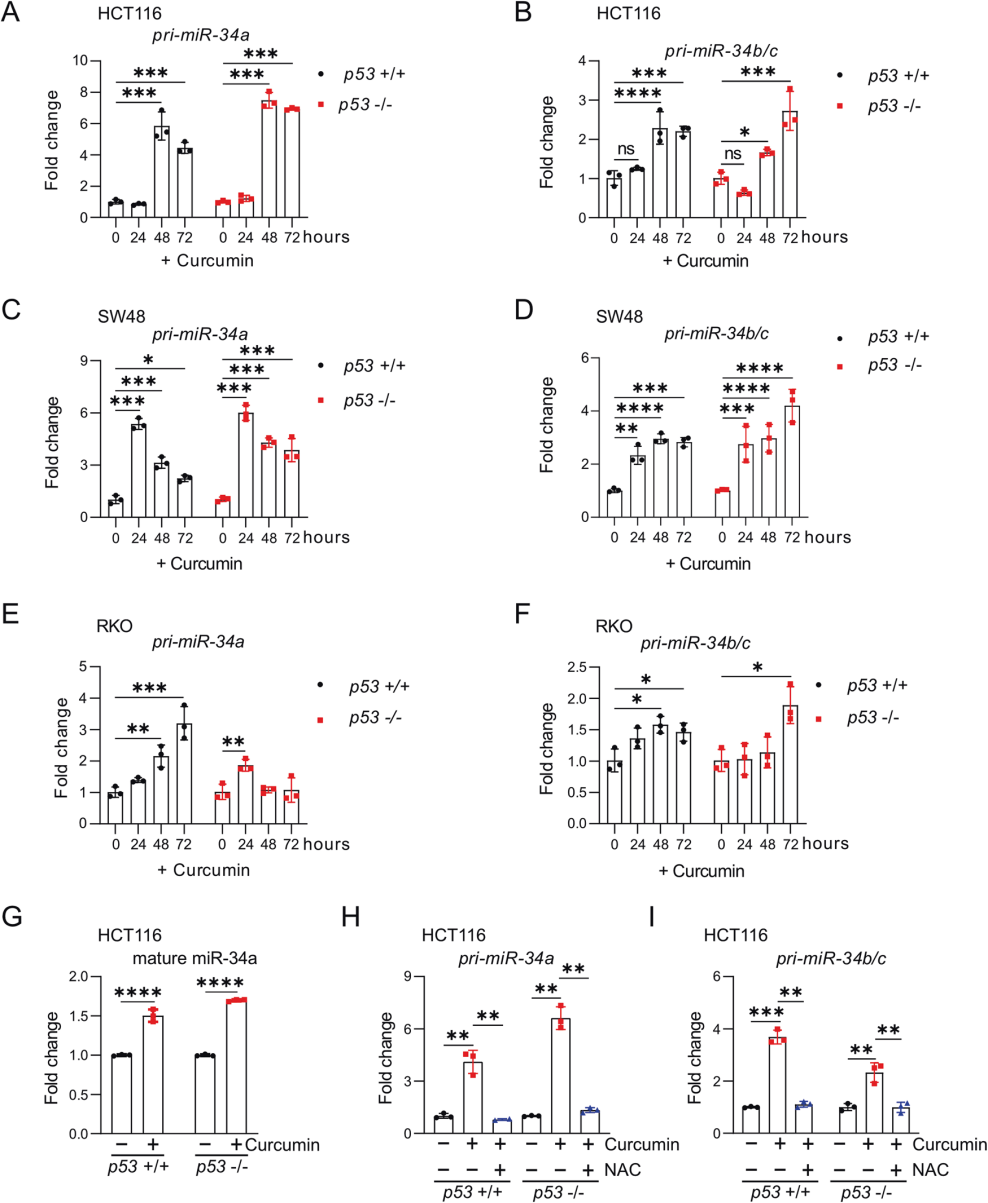
Curcumin up-regulates *miR-34a* and *miR-34b/c* independent of p53. **A**–**F** qPCR analysis of *pri-miR-34a* and *pri-miR-34b/c* expression after treatment of the indicated cells with 15 μM curcumin for the indicated periods. **G** qPCR analysis of mature miR-34a expression in HCT116 cells after treatment with 15 μM curcumin for 48 hours. **H**, **I** qPCR analyses of *pri-miR-34a* and *pri-miR-34b/c* expression in HCT116 cells after treatment with curcumin and/or NAC for 48 hours. In (**A**)–(**I**) mean values ± SD are shown (n = 3). **P <  0.01, ***P <  0.001, ****P <  0.0001.

As indicated in the introduction, previously published observations indicating that miR-34a is induced by curcumin sparked our interest in curcumin. Here we determined whether p53 is required for the induction of *miR-34* genes by curcumin in a set of *p53*-deficient, isogenic CRC cell lines. Interestingly, curcumin induced the expression of the primary *pri-miR-34a* and *pri-miR-34b/c* transcripts in HCT116 cells independent of their *p53* status (Figs. 3A, B, and  S4A, B). Similar results were obtained in the CRC cell lines SW48 and RKO (Figs. 3C–F, and S4C-F). The levels of mature miR-34a were also up-regulated by curcumin in *p53-*proficient and *p53-*deficient HCT116 cells (Figs. 3G and S4G). Since the induction of *pri-miR-34a* and *pri-miR34b/c* by curcumin was prevented by treatment with NAC, it was mediated by ROS (Fig. 3H, I). Taken together, these results showed that curcumin induces *miR-34a* and *miR-34b/c* expression in a *p53*-independent and ROS-dependent manner in CRC cell lines.

### Curcumin-induced NRF2 directly activates *miR-34a* and *miR-34b/c*

**Fig. 4:**
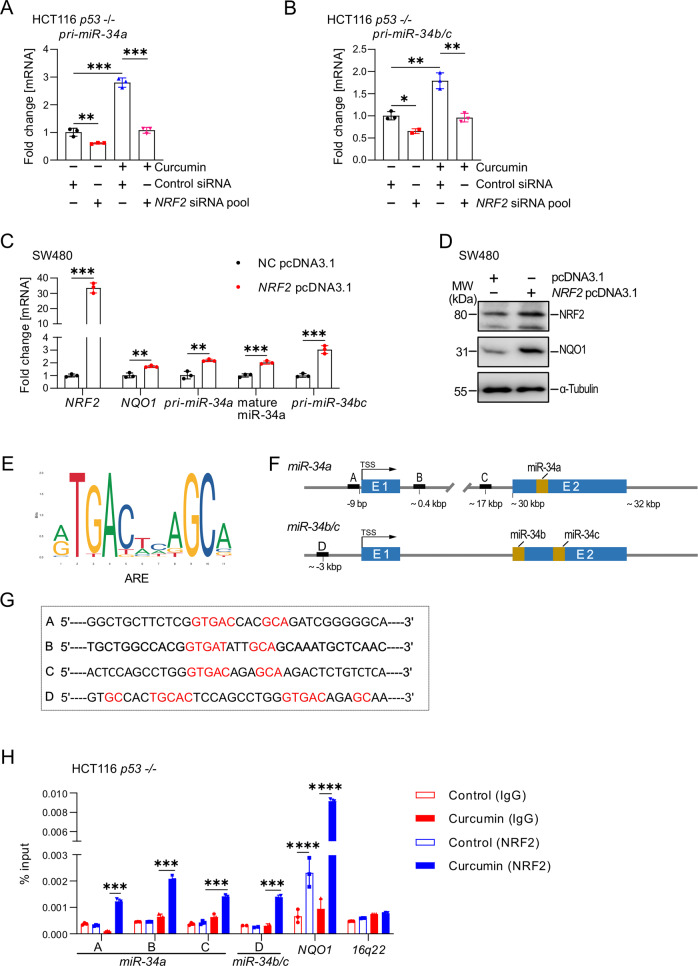
Curcumin-induced NRF2 directly transactivates *miR-34a* and *miR-34b/c*. **A**, **B** qPCR analysis of A. *pri-miR-34a* and B. *pri-miR-34b/c* expression in HCT116 cells treated as indicated for 48 hours. **C** qPCR analysis of indicated mRNAs in SW480 cells after transfection with empty pcDNA3.1 or *NRF2* pcDNA3.1 vectors for 72 hours. **D** Western blot analysis of NRF2 and NQO1 proteins in SW480 cells after transfection with pcDNA3.1 and *NRF2* pcDNA3.1 for 72 hours. **E** ARE consensus sequence defined as 5ʹ-A/G TGA C/G NNNGC A/G-3ʹ, where “N” represents any nucleotide according to jaspar.genereg.net (MA0150.1). **F** Map of human *miR-34a and miR-34b/c* genomic regions with indicated NRF2 binding sites. **G** Sequences of NRF2 binding sites within the *miR-34a* and *miR-34b/c* genomic regions. **H** qChIP analysis of NRF2 occupancy at the human *miR-34a* and *miR-34b/c* genomic regions in HCT116 *p53* −/− cells after treatment with curcumin or DMSO for 48 hours. *NQO1* and *16q22* served as positive and negative controls, respectively. In (**A**)–(**C**), and (**H**) mean values ± SD are shown (n = 3). **P <  0.01, ***P <  0.001.

Next, we hypothesized that NRF2, which is activated by curcumin-induced ROS as shown above, may represent a direct inducer of *miR-34a* and *miR-34b/c* expression. Indeed, suppression of *NRF2* by a pool of 4 different siRNAs prevented the induction of *pri-miR-34a* and *pri-miR-34b/c* expression by curcumin in *p53*-deficient HCT116 cells (Figs. 4A, B, S4A, and S4B). Conversely, ectopic *NRF2* expression increased *pri-miR-34a* and *pri-miR-34b/c* expression, as well as mature miR-34a, *NQO-1* mRNA and protein expression in SW480 cells, which harbour mutant *p53* (Figs. 4C, D). By examining the genomic sequence of the *miR-34a* and *miR-34b/c* promoter regions, we identified three potential NRF2 binding sites (TGAG/CnnnGC), so-called ARE (Antioxidant Response Elements), in the *miR-34a* locus and one potential NRF2 binding site in the *miR-34b/c* locus (Figs. 4E–G). A qChIP assays confirmed enhanced NRF2 occupancy at the four ARE sites within the *miR-34a* and *miR-34b/c* promoters after curcumin treatment for 48 hours (Fig. 4H). Therefore, we concluded that curcumin induces the expression of the *miR-34a* and *miR-34b*/c genes by activating NRF2, which directly binds to the *miR-34a* and *miR-34b/c* promoters and induces their transcription. Notably, this mode of *miR-34* activation is p53-independent.

### *miR-34a* and *miR-34b/c* mediate the effects of curcumin on CRC cells

**Fig. 5:**
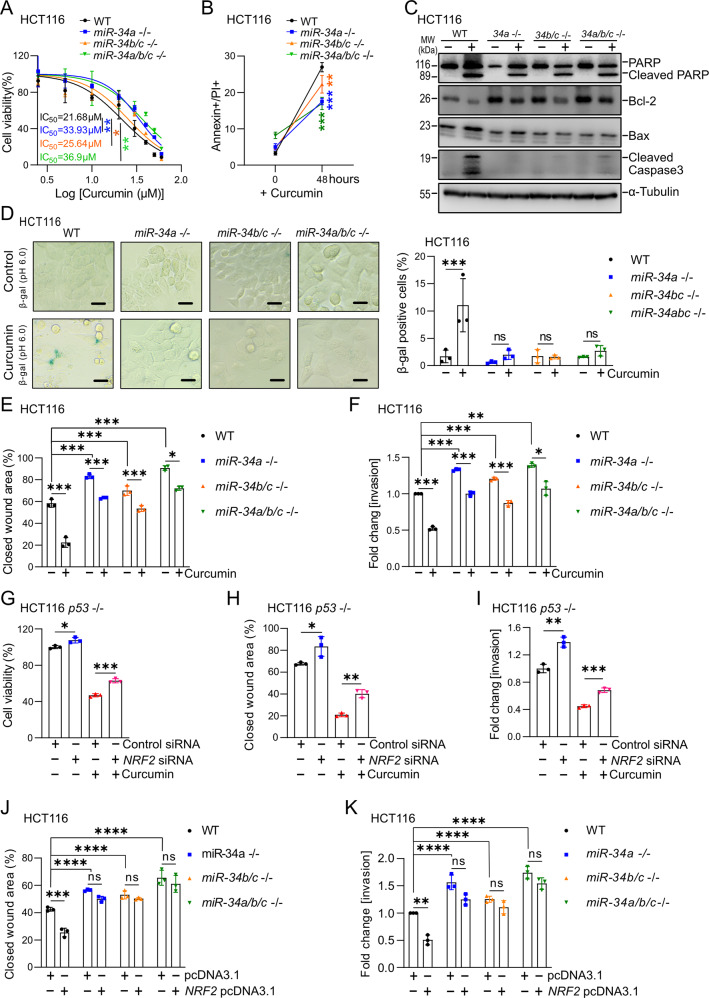
*miR-34a* and *miR-34-b/c* mediate the effects of curcumin on CRC cells. **A** The indicated cell lines were treated with increasing concentrations of curcumin for 48 hours. IC_50_ was determined by MTT assays. **B** Analysis of apoptosis after treatment with 15 μM curcumin for 48 hours determined by Annexin V-FITC and PI staining. **C** The expression of cleaved PARP, Bcl-2, Bax, and cleaved caspase 3 after treatment with 15 μM curcumin for 48 hours was determined by Western blot analysis. **D** Detection of senescent cells after curcumin treatment for 48 hours by pH 6 β-gal staining. **E** Evaluation of migration by wound healing assay 24 hours after treatment with curcumin. **F** Analysis of invasion using Boyden chamber assays 48 hours after treatment with curcumin or DMSO. G. Analysis of cell viability in HCT116 *p53*-deficient cells by MTT assay after transfection *NRF2* siRNA pool or control siRNA pool 24 hours with curcumin for 48 hours. **H** Wound healing assay in HCT116 *p53*-deficient cells treated with curcumin for 24 h after transfection with *NRF2*-specific siRNA pool or control siRNA pool. **I** Invasion assay of HCT116 *p53*-deficient cells exposed to curcumin for 48 hours after transfection with *NRF2*-specific siRNA pool or control siRNA pool. **J** Evaluation of migration by wound healing assay 24 hours after transfection with pcDNA3.1 or *NRF2* pcDNA3.1. **K** Analysis of invasion using Boyden chamber assays after transfection with pcDNA3.1 or *NRF2* pcDNA3.1. In panels **A**, **B**, and **D**–**H** mean values ± SD (n = 3) are shown. *P <  0.05, **P  <  0.01, ***P  <  0.001, ****P <  0.0001.

To determine whether the induction of *miR-34a* and *miR-34b/c* expression is required for the effects of curcumin, we employed isogenic HCT116 cells that were rendered deficient for *miR-34a* and/or *miR-34b/c* genes using a CRSPR/CAS9 approach (their generation will be described elsewhere). The viability of *miR-34a*- or *miR-34a/b/c*-deficient HCT116 cells was significantly higher than *miR-34*-proficient HCT116 cells after treatment with curcumin (Fig. 5A). *MiR-34b/c*-deficient cells showed an intermediate gain of viability when compared to *miR-34a/b/c*-proficient HCT116 cells. In agreement, *miR-34a/b/c*-deficiency resulted in a decreased enhancement of apoptosis by curcumin when compared to *miR-34a/b/c*-proficient HCT116 cells (Figs. 5B and S6A). In *miR-34a/b/c*-proficient HCT116 cells, curcumin treatment induced strong cleavage of caspase 3, whereas cleaved caspase 3 was not detectable in *miR-34*-deficient cells (Fig. 5C). In *miR-34a/b/c*-proficient HCT116 cells, curcumin suppressed the expression of the anti-apoptotic protein Bcl-2, a known target of miR-34 [28], and induced the expression of the pro-apoptotic protein BAX (Fig. 5C). HCT116 cells with single or combined deletion of *miR-34a* and *miR-34b/c* displayed elevated expression of Bcl-2 and after treatment with curcumin the expression of Bcl-2 exceeded the levels detected in untreated WT cells. This effect was most pronounced in the *miR-34a/b/c*-deficient HCT116 cells. Furthermore, the induction of BAX by curcumin was diminished in HCT116 cells with *miR-34a/b/c*-deficiency (Fig. 5C). Therefore, it is conceivable that the repression of Bcl-2 by miR-34a/b/c contributes to the induction of apoptosis by curcumin.

*miR-34a*, *miR-34b/c*, and *miR-34a/b/c*-deficient cells exhibited no increase in senescence after curcumin treatment as determined by detection of β-galactosidase activity at pH 6.0, whereas WT HCT116 cells showed a signficiant increase in senescence (Fig. 5D). Finally, the curcumin-induced suppression of migration and invasion was significantly lower in *miR-34a-, miR-34b/c-*, and *miR-34a/b/c*-deficient cells than in *miR-34*-proficient HCT116 cells (Figs. 5E, F, S6B, and S6C). In all these assays cells with combined inactivation of *miR-34a* and *miR-34b/c* showed the strongest resistance to curcumin. Knockdown of *NRF2* by siRNAs partially prevented the curcumin-induced suppression of cell viability (Fig. 5G), migration (Figs. 5H and S7A), and invasion (Figs. 5I and S7B). Furthermore, ectopic expression of *NRF2* repressed the migration and invasion of *miR-34*-proficient HCT116 cells (Figs. 5J, K, S7C, and S7D) in the absence of treatment with curcumin. However, in *miR-34a/b/c*-deficient HCT116 cells ectopic NRF2 had no effect on migration and invasion, demonstrating that *miR-34a* and *miR-34b/c* are required mediators of NRF2 function. Taken together, our results demonstrate that the NRF2-mediated activation of *miR-34a* and *miR-34b/c* genes is a required mediator for the effects on curcumin on apoptosis, senescence, migration and invasion.

### The induction of *miR-34a/b/c* by H_2_O_2_ and tBHP is mediated by NRF2

**Fig. 6:**
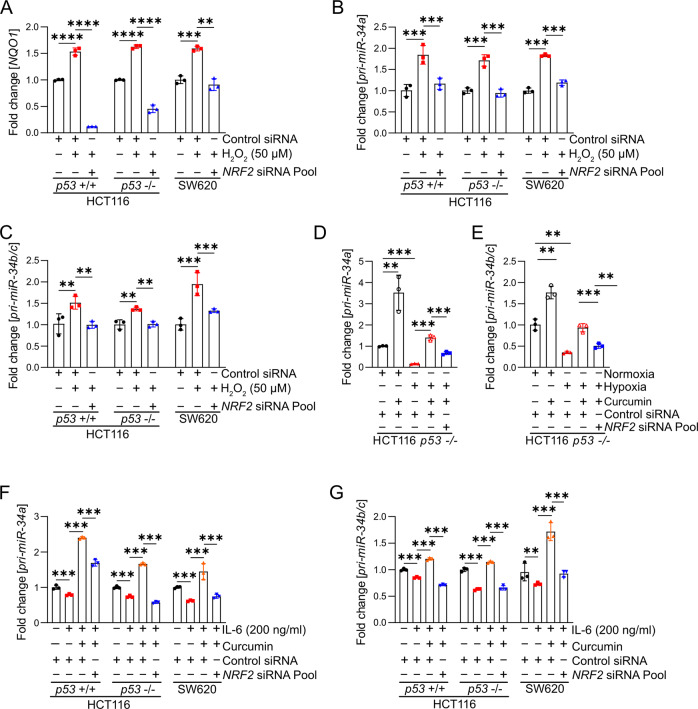
Effects of the NRF2 axis on regulation of *miR-34a/b/c* by H_2_O_2_, curcumin, hypoxia and IL-6. **A**–**C** Expression of *NQO1* (A), *pri-miR-34a* (B), and *pri-miR-34b/c* (C) after treatment with H_2_0_2_ and transfection with control or NRF2 siRNA for 24 hours. **D**, **E** Expression of *pri-miR-34a* (**D**) and *pri-miR-34b/c* (**E**) in *p53*-deficient HCT116 cells after the indicated transfections/treatments. **F**, **G** Expression of *pri-miR-34a* (F) and *pri-miR-34b/c* (G) in indicated cells after transfection with control or *NRF2* siRNA and treatment with curcumin and/or IL-6 (200 ng/ml) for 48 hours. In (**A**)–(**G**) mean values ± SD are shown (n = 3)*P <  0.05, **P <  0.01, ***P <  0.001, ****P  <  0.0001.

It has been previously shown, that the ROS induced by treatment with H_2_O_2_ induces the expression of miR-34a [29]. Here, we determined whether this regulation is mediated by NRF2. First, we analysed cell viability of CRC cell lines after treatment with H_2_O_2_ and determined IC_50_ values of 92.61, 48.35, and 61.52 µM for *p53*-proficient HCT116 cells, *p53*-deficient HCT116 cells, and SW620 cells, which express mutant p53, respectively (Figs. S8A and S8B). Treatment of *p53*-proficient and *p53*-deficient HCT116 cells as well as SW620 cells with H_2_O_2_ or tBHP resulted in the induction of the NRF2 target gene *NQO1*, indicating that H_2_O_2_ and tBHP activate NRF2 (Figs. 6A and S8C). Importantly, H_2_O_2_ and tBHP induced the expression of *miR-34a/b/c*, which was prevented by siRNA-mediated suppression of NRF2 (Figs. 6B, C, S8D, and S8E).

### The suppression of *miR-34a/b/c* by hypoxia or IL-6 is reversed by curcumin-induced NRF2

We have previously demonstrated that hypoxia [20] and IL-6-mediated STAT3 activation [18] repress *miR-34a/b/c* expression in *p53*-deficient CRC cells. To determine whether curcumin could reverse the hypoxia-mediated suppression of *miR-34a/b/c*, we cultured *p53*-deficient HCT116 cells under hypoxia and treated them with curcumin. Indeed, the hypoxia-mediated suppression of *miR-34a/b/c* expression was completely reversed by curcumin (Figs. 6D, E). However, this reversion was significantly weaker when *NRF2* was suppressed by siRNAs (Fig. 6D, E), indicating that this effect of curcumin is largely mediated by NRF2 activation. Previous studies showed that IL-6/STAT3 signalling suppresses *miR-34a* expression via direct binding of STAT3 to the *miR-34a* promoter region [20]. Notably, the IL-6-mediated suppression of *miR-34a* and also that of *miR-34b/c* was completely reversed by treatment with curcumin (Fig. 6F, G). However, this was not the case when NRF2 was suppressed by siRNA (Fig. 6F, G), indicating that this effect of curcumin was mediated by NRF2.

### Curcumin induces MET and inhibits lung-metastases formation via inducing miR-34a

**Fig. 7:**
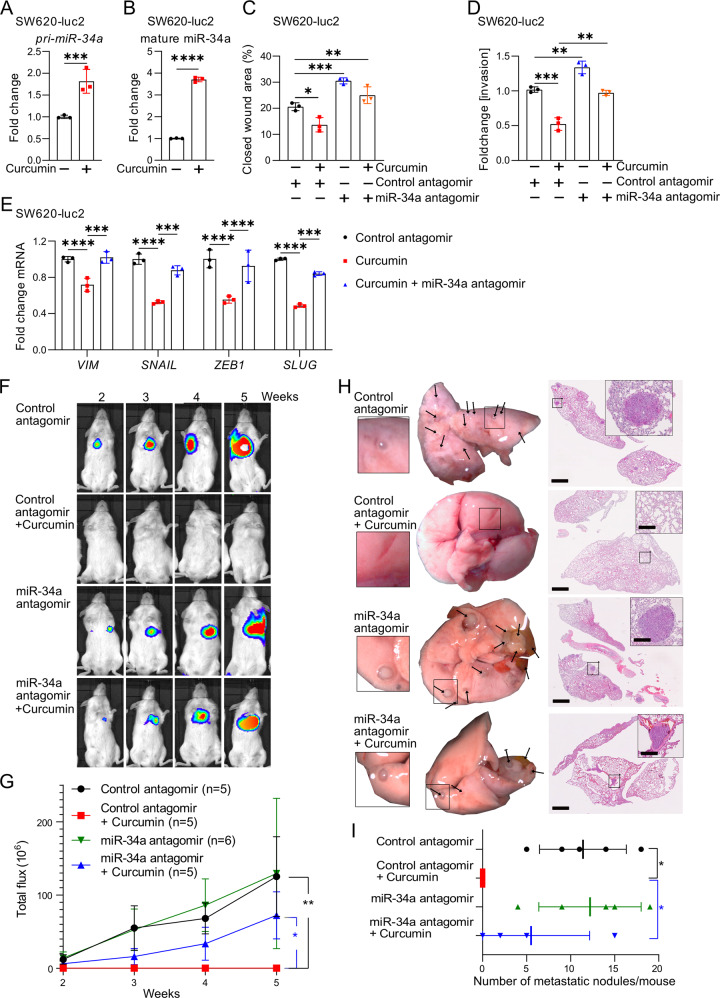
Curcumin induces MET and inhibits lung-metastases formation via inducing miR-34a. **A**, **B** Expression of *pri-miR-34a* (A) and mature miR-34a (B) in SW620-luc2 cells treated with curcumin or DMSO for 48 hours. **C** Analysis of SW620-luc2 cell migration after transfection with miR-34 antagomirs and/or curcumin for 72 hours. **D** Analysis of SW620-luc2 cell invasion after transfection with miR-34 antagomirs and/or curcumin for 72 h. **E** qPCR analyses of EMT-related genes in SW620-luc2 cells *72* hours after the indicated treatments/transfections. **F**–**I** Formation of lung metastases by SW620-Luc2 cells, which were treated as indicated for 48 hours and then injected into tail-vein of NOD/SCID mice. Representative images of luciferase signals at the indicated time points after xenografting (**F**) and the quantification of total photon flux (**G**). **H** right: representative lungs 5 weeks after tail vein injection. Arrows indicate metastatic tumor nodules. Left: representative images of H&E-stained resected lungs. Scale bar: 500 μm; 50 μm (insert). **I**. Quantification of metastatic nodules in the lungs of indicated mice. In (**A**)–(**E**) mean values ± SD (n = 3) are shown. *P <  0.05, **P <  0.01, ***P <  0.001, ****P  <  0.0001.

Next, we characterized the effect of curcumin on SW620-Luc2 cells, which stably express luciferase, since we intended to study these cells after transplantation into mice. Curcumin inhibited the viability of SW620-Luc2 cells in a dose-dependent manner (Fig. S9A). The IC_50_ was 14.49 μM as determined by an MTT assay. This concentration of curcumin was used subsequently. The expression of *pri-miR-34a* and mature miR-34a was upregulated after exposure of SW620-Luc2 cells to curcumin for 48 hours (Fig. 7A, B). Mature miR-34b and miR-34c were expressed at very low levels in SW620-Luc2 cells when compared to mature miR-34a, also after exposure to curcumin (Fig. S9B). Therefore, we focussed on the analysis of the role of miR-34a for the effects of curcumin on SW620-Luc2 cells. Our previous studies had shown that *miR-34a* critically contributes to p53-induced mesenchymal-epithelial-transition (MET) in CRC cells [17, 30]. Therefore, we asked whether curcumin induced miR-34a may mediate MET. Indeed, treatment of SW620-Luc2 cells with curcumin resulted in repression of the mesenchymal markers *Vimentin* (*VIM*), *SNAIL*, *SLUG*, and *ZEB1* (Fig. 7E). Also this inhibitory effect of curcumin was abolished by miR-34a-specific antagomirs. Curcumin also suppressed invasion and migration, which presumably is a functional consequence of MET, in SW620-Luc2 cells in a miR-34a-dependent manner, as determined by silencing of miR-34a using antagomirs (Figs. 7C, D, S9C, and 9D). Finally, we performed mouse xenograft experiments to determine whether curcumin affects the capacity of CRC cells to form lung metastases. Therefore, we treated SW620-Luc2 cells ex vivo with curcumin or/and miR-34a-specific antagomirs for 48 hours. Subsequently, these cells were injected into the tail veins of NOD/SCID mice to assess the formation of lung metastasis. Longitudinal, non-invasive imaging showed that the treatment of SW620-Luc2 cells with curcumin completely abrogated metastasis formation within 5 weeks after injection (Fig. 7F, G). However, concomitant inhibition of miR-34a by antagomirs partially restored metastasis formation after curcumin treatment (Fig. 7F, G). Five weeks after injection, resected lungs were devoid of macroscopically visible metastases in mice injected with curcumin-treated SW620-Luc2 cells (Fig. 7H). Haematoxylin and eosin (H&E) staining confirmed the absence of metastatic nodules in mice injected with curcumin-treated cells (Fig. 7H, I). However, SW620-luc2 cells concomitantly treated with miR-34a-antagomirs and curcumin, formed lung-metastases (Fig. 7H, I). In summary, these results show that curcumin inhibits metastases formation via inducing *miR-34a*.

### *p53* and *miR-34a/b/c* modulate sensitivity towards curcumin and/or 5-FU

**Fig. 8:**
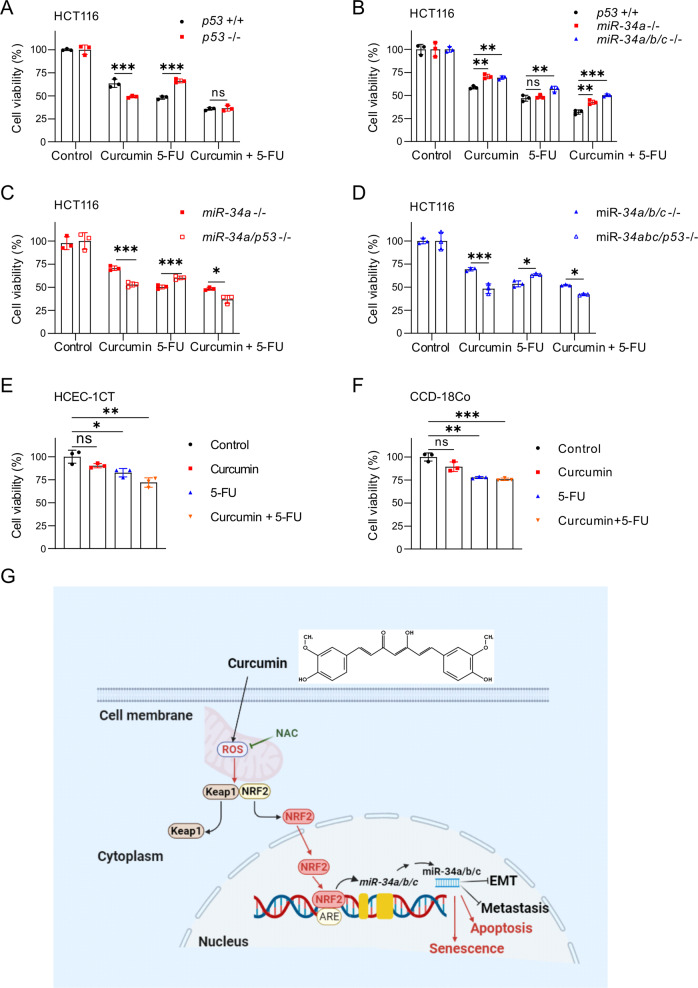
*p53* and *miR-34a/b/c* modulate sensitivity towards curcumin and/or 5-FU. **A**–**D** HCT116 cells with the indicated genotypes were exposed to curcumin (15 μM), 5-FU (2 mg/L), or curcumin combined with 5-FU. After 48 hours cell viability was determined by MTT assays. **E**, **F** The viability of HCEC-1CT and CCD-18Co cells was determined by MTT assay after the indicated treatments for 48 hours. G. Schematic model of the findings obtained in this study.

Finally, we investigated the effect of curcumin on CRC cell viability in combination with the chemotherapeutic drug 5-fluorouracil (5-FU), which is widely used for the treatment of CRC. For this we used clinically relevant and tolerable concentrations of 5-FU (2 mg/L) and curcumin (15 µM) [31, 32]. Combined treatment of HCT116 cells with curcumin and 5-FU showed a stronger suppression of cell viability when compared to single treatment with either compound (Fig. 8A). Compared to *p53*-proficient cells, *p53*-deficient cells were more sensitive to curcumin but more resistant to 5-FU. However, no difference was observed between *p53*-deficient and *p53*-proficient cells when treated with curcumin and 5-FU (Fig. 8A). Compared to wt cells, *miR-34a*- and *miR-34a/b/c*-deficient cells were more resistant to curcumin, but only marginally more resistant to 5-FU. However, *miR-34a-* and *miR-34a/b/c*-deficiency resulted in a markedly higher resistance to the combination of curcumin and 5-FU (Fig. 8B). Additional deletion of *p53* in *miR-34a-* or *miR-34a/b/c*-deficient cells increased their resistance to 5-FU. Interestingly, inactivation of *p53* reversed the resistance of *miR-34a-* or *miR-34a/b/c*-deficient cells to curcumin and the combination of curcumin and 5-FU (Fig. 8C, D). Human colonic epithelial cells (HCEC-1CT) and human intestinal fibroblasts (CCD-18Co cells) were less sensitive to curcumin and/or 5-FU than HCT116 cells (Fig. 8E, F). Taken together, these results suggest that curcumin may enhance the therapeutic effects of 5-FU on CRC cells. Interestingly, this effect is most pronounced in cells lacking *p53* and *miR-34a/b/c*, which are also known to give rise to CRCs with the poorest prognosis [21].

## Discussion

Here we identified a new regulatory connection that explains how curcumin up-regulates the expression of *miR-34a* and *miR-34b/c* in a p53-independent manner (for a summary see Fig. 8G). Furthermore, we show that *miR-34a* and *miR-34b/c* mediate senescence and apoptosis, and inhibition of migration, invasion and metastasis of CRC cells after exposure to curcumin. Interestingly, the induction of *miR-34a* and *miR-34b/c* by curcumin was dominant over their repression by exposure to IL-6 or hypoxia. Therefore, curcumin may re-activate *miR-34* expression, which is repressed due to signals generated by the tumor microenvironment. In addition, inhibition of miR-34a function in CRC cells prevented the curcumin-mediated suppression of lung metastases formation of CRC cells transplanted into immune-incompetent mice. Taken together, these results establish a central role of the ROS/KEAP1/NRF2/*miR-34a/b/c* axis in mediating the tumor suppressive effects of curcumin.

Here, exposure of CRC cells to curcumin induced ROS, which activated the KEAP1/NRF2-pathway. Under normal conditions, NRF2 is rapidly degraded and sequestered in the cytoplasm by a KEAP1-CUL3-RBX1 complex [25]. In the presence of oxidative stress, electrophiles and ROS react with cysteine residues within the KEAP1 protein, which alters the conformation of KEAP1 and results in the release of NRF2 from the KEAP1-CUL3-RBX1 complex. Subsequently, NRF2 translocates to the nucleus, where it binds to ARE motifs in the vicinity of promoters of genes that encode mediators of the antioxidant response and activates these. Here, *miR-34a/b/c* were not only induced by curcumin, but also by H_2_O_2_ and tBHP in an NRF2-dependent manner. Therefore, our results imply that the *miR-34a* and *miR-34b/c* genes represent an integral part of the oxidative stress response program controlled by NRF2. The different processes regulated by ROS-induced miR-34, such as cell cycle progression, apoptosis, senescence, autophagy, EMT and migration, may allow cells to appropriately react to oxidative stress. The loss of *miR-34a/b/c* in cancer cells may allow proliferation and survival under the adverse conditions present in the tumor microenvironment, which includes high levels of ROS. Here, the antioxidant N-acetylcysteine suppressed the induction of apoptosis by curcumin. Therefore, the accumulation of ROS presumably mediates curcumin-induced apoptosis. The diketone group of curcumin conjugates with glutathione-SH, leading to the depletion of the glutathione pool and exhaustion of the antioxidant defense system in cells [33]. Curcumin has both antioxidative and pro-oxidative properties depending on the dose and cell types. At very low concentrations ( ≤ 1 μM) curcumin functions as an antioxidant in non-cancerous cells [34]. However, in cancer cells higher concentrations (5-10 μM) of curcumin induce autophagy and ROS production [35, 36]. In addition, concentrations of curcumin beyond 5.8 ± 1.6 µM induce endoplasmic reticulum (ER) membrane destabilization [37, 34] and inhibit Ca2 + -ATPase (SERCA) [38], thereby leading to release of Ca2+ causing mitochondrial destabilization and thereby additional release of ROS [39]. Interestingly, the amount of ROS induced by curcumin was higher in *p53*-deficient CRC cells than in *p53*-proficient cells, indicating that p53 suppresses the generation of ROS. p53 was shown to induce the expression of factors, which may mediate this anti-oxidative effect, such as GLS2 [40], TIGAR [41], and p53R2 [42]. GLS2 is a regulator of glutathione (GSH) synthesis, and may thereby facilitate glutamine metabolism lowering intracellular ROS levels after p53 activation [43, 44]. The p53-induced TIGAR protein represses the expression of fructose-2,6-bisphosphate levels in cells, resulting in an inhibition of glycolysis and an overall decrease in intracellular ROS levels [41, 45]. These regulations may explain why curcumin generated more ROS in *p53*-deficient cells when compared to *p53*-proficient HCT116 cells. Importantly, loss of p53 function in tumor cells may sensitize to curcumin-induced apoptosis via allowing an increased generation of ROS. Interestingly, *p53*-deficient cells were more sensitive to curcumin than *p53*-proficient cells. This suggests that CRCs, which display a high frequency of *p53* inactivation by mutation, may be especially sensitive to treatment with curcumin. The mild induction of ROS by curcumin presumably contributes to its hormetic effects [46], which may according to our results, at least in part, be mediated activation of *miR-34a* and *miR-34b/c*.

It was reported that *miR-34a* expression is induced by curcumin in *p53*-deficient cells, but not in *p53*-proficient cells [47]. However, the mechanism remained unknown. Furthermore, another study reported a *p53*-dependent activation of *miR-34a* by curcumin [48]. Our results provide clear evidence that *miR-34a* and *miR-34b/c* are activated by curcumin in a p53-independent manner via the ROS/KEAP1/NRF2/ARE pathway and represent direct targets of NRF2. Besides p53, which activates *miR-34a*, several transcription factors that repress *miR-34a* expression have been described: HIF1α and STAT3 repress *miR-34* genes under conditions of hypoxia and inflammation, which are hallmarks of the tumor micro-environment [18, 20]. The repression of *miR-34a* has been shown to contribute to the invasion and metastasis of CRCs [21]. Since curcumin was able to antagonize the repression of *miR-34a* and *miR-34b/c* by hypoxia or IL6 exposure, a therapeutic treatment with curcumin may therefore reactivate *miR-34* expression in primary CRC and metastases, and thereby suppress progression of CRCs.

We identified *miR-34a* and *miR-34b/c* as central effectors of the ROS/NRF2 pathway which were activated by curcumin as well as by ROS-generating substances, such as H_2_O_2_ and tBHP. Therefore, *miR-34a* and *miR-34b/c* not only mediate the cellular response to activation of the p53 pathway, but also represent integral components of the response to oxidative stress. Furthermore, our results provide a plausible mechanism for the effects that have been ascribed to curcumin in the prevention and therapy of colorectal cancer and other malignancies. The members of the miR-34 family are frequently silenced in colorectal tumors by DNA methylation [16]. There is evidence that curcumin can reactivate CpG methylated genes [49]. Therefore, CpG-methylation of *miR-34a/b/c* is presumably not an obstacle for treatment of CRC with curcumin. Originally this study was intended to determine the mode of action of curcumin during tumor prevention. In that scenario, *miR-34a/b/c* should not be silenced by CpG-methylation. We showed that the anti-tumor effects of curcumin are less pronounced in *miR-34*-deficient cells. Therefore, it will be important to investigate the in vivo effects of curcumin on CRC treatment and prevention with respect to miR-34 expression in the future. For example, future experiments should include the treatment of wt and *miR-34* knockout mouse models of CRC with curcumin and/or chemotherapy. Howells et al. [7] showed in a phase IIa clinical study that the addition of curcumin to FOLFOX treatment significantly improved the progression free and overall survival. The addition of curcumin to cancer therapy is of great interest, since a phase I clinical study showed that the addition of curcumin to FOLFOX treatment is safe and tolerable in patients with metastatic CRC at doses up to 2 grams daily [50]. Moreover, oral consumption of up to 3600 mg curcumin leads to curcumin concentrations in human colorectal mucosa which are in the range of the concentration used in this study [51]. In the future, the findings presented here may be exploited for the development of therapeutic approaches that aim at restoring the tumor suppressive function of the p53/miR-34 pathway.

## Materials and Methods

### Cell culture and treatments

HCT116, RKO, SW48, CCD-18Co, and SW620-Luc2 cell lines were cultured in McCoy’s 5 A medium (Invitrogen, Carlsbad, CA, USA) with 10% fetal bovine serum (FBS) (Invitrogen) containing 100 units/ml penicillin and 0.1 mg/ml streptomycin. HCEC-1CT immortalized human colonic epithelial cells (Evercyte GmbH) were maintained in DMEM supplemented with 2% FBS, 1× N2 Supplement (contains insulin, apo-transferin, and sodium-selenite; Thermo Fisher Scientific), 20 ng/mL epidermal growth factor (EGF; AF-100–15, PeproTech, part of Thermo Fisher Scientific), 1 μg/mL hydrocortisone (Sigma, Merck), and 1× penicillin/streptomycin (Gibco, Thermo Fisher Scientific). For the 5-FU experiment dialyzed FBS (A3382001; Thermo Fisher Scientific). All cells were incubated at 20% O_2_, 5% CO_2_, and 37°C incubator. Curcumin (Sigma-Aldrich, #C1386) was dissolved in DMSO (stock concentration 50 mM) and the working concentration was 15 μM. NAC (Sigma-Aldrich, #A0737) was dissolved in water (stock concentration 100 mM) and the working concentration was 5 mM. IL-6 (Immunotools) was dissolved in water and used at a final concentration of 200 ng/ml. 5-FU (Sigma-Aldrich, # 343922) was dissolved in DMSO (stock concentration 5 mg/ml) and the working concentration was 2 µg/ml. siGENOME Human *NRF2* siRNA SMARTPool (Horizon Discovery, USA) and siGENOME Non-Targeting siRNA Control Pools (Horizon Discovery, USA), miRNA antagomir (hsa-miR-34a-5p inhibitor, Invitrogen, MH11030), and respective negative controls were transfected at a concentration of 10 nM using HiPerFect transfection reagent (Qiagen, Hilden, Germany). Hypoxia (0.5% O_2_) was achieved using a CD210 incubator (Binder, Tuttlingen, Germany).

### MTT assay

Cell viability was analyzed by MTT assays. CRC cells were seeded at a density of 3000 cells per well in 96-well plates. After 24 hours, cells were treated with curcumin at the indicated concentrations for 48 hours, followed by addition of 10 μl MTT solution (5 mg/ml) per well and incubation for 4 hours. Cells were lysed with 100 μl DMSO per well for 15 min to release the resulting formazan according to Twentyman et al. [52], and analyzed by measuring the absorbance at 570 nm by a Varioscan system (Thermo Fisher).

### RNA isolation and qPCR analysis

Total cellular RNA was isolated and purified from CRC cell lines according to manufacturer’s instruction (High Pure RNA Isolation Kit; Roche). 1ug of total RNA was used for reverse transcription with Verso cDNA Synthesis Kit (Termo Fisher Scientifc, Waltham, MA, USA). Quantitative real-time polymerase chain reaction (qPCR) was performed using Fast SYBR Green Master Mix (Applied Biosystems, Foster City, CA) on a LightCycler 480 (Roche) system. qRT-PCR data was normalized to *GAPDH* or β-*actin* and analyzed with the ΔΔCt method [53]. Primer sequences information are provided in Table S1.

### Immunofluorescence analysis by confocal laser-scanning microscopy

Cells were cultured and treated with curcumin on glass cover slides, fixed in 4% paraformaldehyde/PBS for 15 min, permeabilized in 0.2% Triton X-100 for 5 min, and then blocked in 1% BSA/PBS for 1 h at room temperature. Next, cells were incubated with NRF2 primary antibody (D1Z9C, #12721, Cell Signaling Technology) for 1 h at room temperature, washed 3 time with PBS-Tween and then incubated with cy3-labelled secondary antibody (ab6939, Abcam). Chromatin was stained with DAPI (Roth). The images were acquired by confocal laser scanning microscopy (CLSM) using the LSM700 microscope with a Plan Apochromat 20×/0.8 M27 objective and ZEN 2009 software (Zeiss).

### Chromatin immunoprecipitation

HCT116 *p53*-/- were treated with curcumin for 48 hours to activate NRF2 before cross-linking. ChIP experiments were performed using the iDeal ChIP-qPCR kit (Diagenode, Belgium) according to manufacturer’s instructions. The sequences of qChIP primers are provided in Table S2.

### Detection of cellular ROS

Reactive oxygen species (ROS) were detected by DCFDA/H2DCFDA staining. In brief, HCT116 cells were seeded at a density of 10^4^ cells per well in 96 well plates. After 24 hours, curcumin or DMSO was added for 48 hours. The positive control tBHP was added to HCT116 cells for 4 hours before staining. After removal of medium 1X Buffer (100 µl/well) was added. After removal of the 1X Buffer, cells were stained with DCFDA solution (100 µl/well) for 45 min at 37 °C in the dark. After removal of the DCFDA solution, plates were analyzed immediately on a fluorescence plate reader at Ex/Em = 485/535 nm. Image J software was used to analyze the images and compared the control group.

### Metastases formation in a xenograft mouse model

SW620 cells stably expressing *Luc2* were described previously [30]. Cells were injected into the lateral tail vein of NOD/SCID mice using 25-gauge needles (4 × 10^6^ cells /0.2 ml) in HBSS. From the second week onwards, mice were injected i.p. with D-luciferin (150 mg/kg) and imaged for 5 minutes using the IVIS Illumina System (Caliper Life Sciences) in weekly intervals. Five weeks after tail vein injection, mice were sacrificed and examined for lung metastases using H&E staining. Animal experimentations and analyses were approved by the Government of Upper Bavaria, Germany (55.2-2532.vet_02-18-57).

### Statistical analysis

The statistical differences between two groups were calculated using a Student’s t test (two-tailed; unpaired). When more than 2 groups were compared, the one-way analysis of variance (ANOVA) with the Tukey multiple comparison post-test was used. with p  <  0.05 considered significant. Asterisks generally indicate: *p  <  0.05, **p  <  0.01 and ***p  <  0.001, n.s. = not significant. Additional method descriptions can be found as supplementary information.

## Supplementary information

Supplemental Material

Reproducibility-checklist

## Data Availability

All data, analytic methods, and study materials will be made available to other researchers upon reasonable request.
