# Supplementary material for: Curcumin activates a ROS/KEAP1/NRF2/miR-34a/b/c cascade to suppress colorectal cancer metastasis
Source: Cell Death Differ. 2023 May 20;30(7):1771–85. doi: 10.1038/s41418-023-01178-1 (PMC10307888; doi:10.1038/s41418-023-01178-1)
Supplement: Supplementary file 1 — Supplemental Material [file 41418_2023_1178_MOESM1_ESM.pdf]

Supplementary Material

Curcumin activates a ROS/KEAP1/NRF2-*miR-34a/b/c* cascade to suppress colorectal cancer metastases

Chunfeng Liu, Matjaz Rokavec, Zekai Huang and Heiko Hermeking

Inventory of Supplementary Material:

- **Supplementary Figure 1**
  Related to Figure 1
  p53-independent effects of Curcumin on CRC cells

- **Supplementary Figure 2**
  Related to Figure 2
  Curcumin induced ROS generation in CRC cells

- **Supplementary Figure 3**
  Related to Figure 2
  Curcumin activates NRF2 via ROS in CRC cells

- **Supplementary Figure 4**
  Related to Figure 3
  Curcumin up-regulates *miR-34a* and *miR-34b/c* independent of p53

- **Supplementary Figure 5**
  Related to Figure 4
  NRF2 directly induces the expression of *miR-34a* and *miR-34b/c*

- **Supplementary Figure 6**
  Related to Figure 5
  Curcumin promotes apoptosis and inhibits tumor cell growth via up-regulation of *miR- 34* expression

- **Supplementary Figure 7**
  Related to Figure 5
  Curcumin repressed migration and invasion via activated *NRF2*/*miR-34* axis.

- **Supplementary Figure 8**
  Related to Figure 7
  The induction of *miR-34* by tBHP is mediated by NRF2

- **Supplementary Figure 9**
  Related to Figure 7
  Curcumin inhibits lung-metastases formation via inducing *miR-34a*

- **Supplementary Figure:**
  Original Western blots (Uncropped membranes)

- **Supplementary Table 1**
  Oligonucleotides used for qPCR

- **Supplementary Table 2**
  Oligonucleotides used for qChIP

- **Supplementary Table 3**
  List of antibodies

- **Supplementary Methods:**

  - Assessment of cell proliferation by real-time impedance measurement
  - Cell cycle analysis by flow cytometry
  - Detection of apoptosis by flow cytometry
  - Western blot analysis
  - Beta-galactosidase (β-gal) staining
  - Modified Boyden-chamber assay

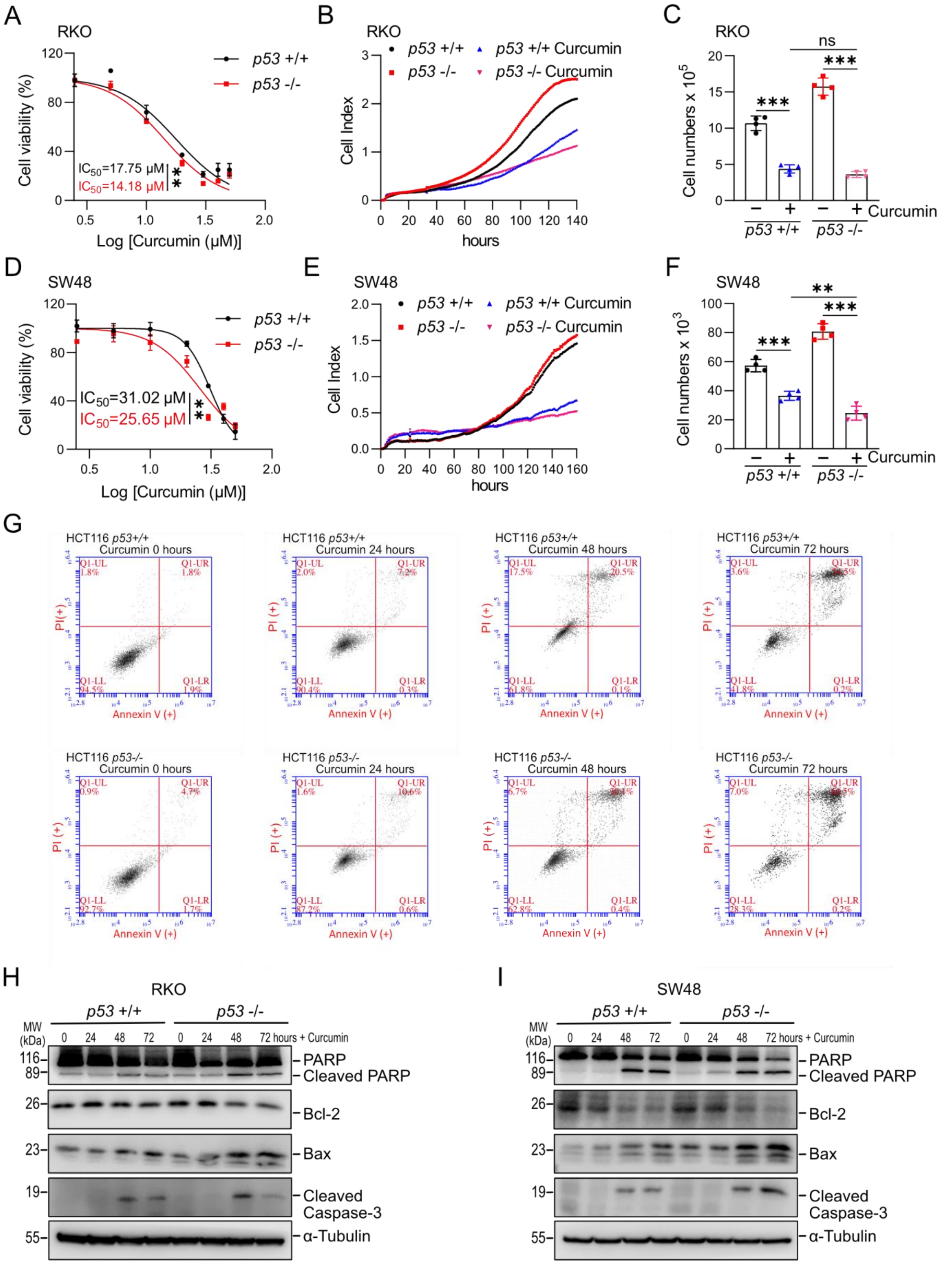

Figure S1: *p53*-independent effects of Curcumin on CRC cells

A. The RKO *p53* +/+ and *p53* -/- cells were exposed to indicated concentrations of curcumin for 48 hours. MTT assays were used to determine the IC_{50} values. B and C.

Cell proliferation of RKO was analyzed by measuring the impedance with xCELLigence after treatment with curcumin (15 μM). The number of cells were counted at the last time point. D. SW48 cells were treated with curcumin, and IC_{50} values were determined via MTT assay. E and F. Cell proliferation was detected by measuring impedance with xCELLigence after treatment with curcumin (15 μM). The number of cells were counted at the last time point. G. HCT116 *p53 +/+* and *p53 -/-* cells were treated with curcumin (15 μM) for the indicated time point following by Annexin V-FITC and PI staining to detect apoptotic cells. H and I. Expression of the indicated proteins was detected by western blot after exposure to curcumin at different time points in RKO and SW48 cells. In panels (A, B, D, E, and G) the mean ± SD (n=3) is provided. In panels (C and F) the mean ± SD (n=4) is provided. * $P < 0.05$, ** $P < 0.01$, *** $P < 0.001$.

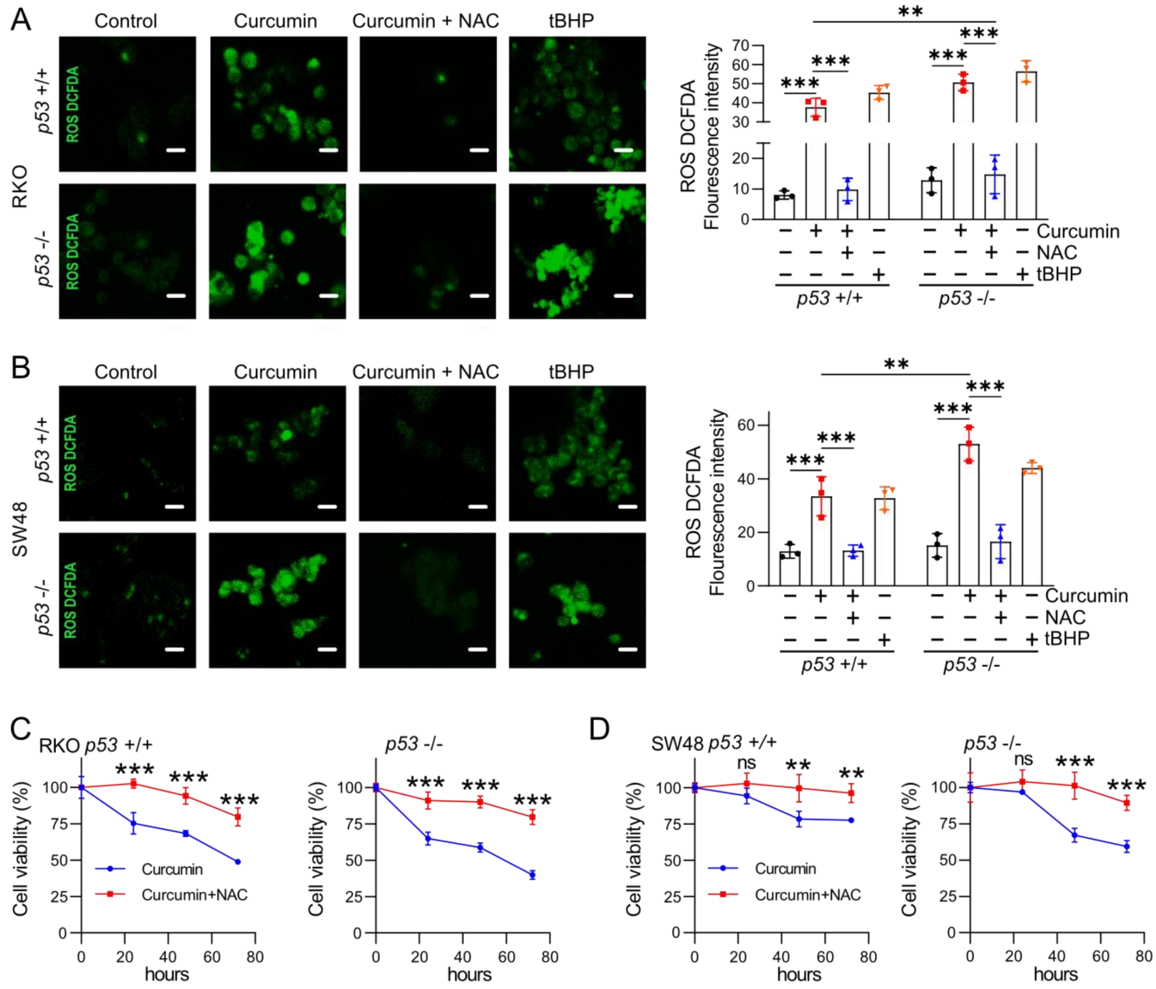

Figure S2: Curcumin induced ROS generation in CRC cells

A. Analysis of ROS formation in RKO cells (A) and SW48 cells (B) treated as indicated. Left panel: for representative IF pictures. Scale bar: 100 µM. Right panel: quantification of fluorescence intensity. Cell viability of RKO (C) and SW48 (D) Cells were analyzed after treatment with curcumin or curcumin combined with NAC at the indicated time points. In panels (A-D) the mean ± SD (n=3) is provided. ** P < 0.01, *** P < 0.001, ns = not significant.

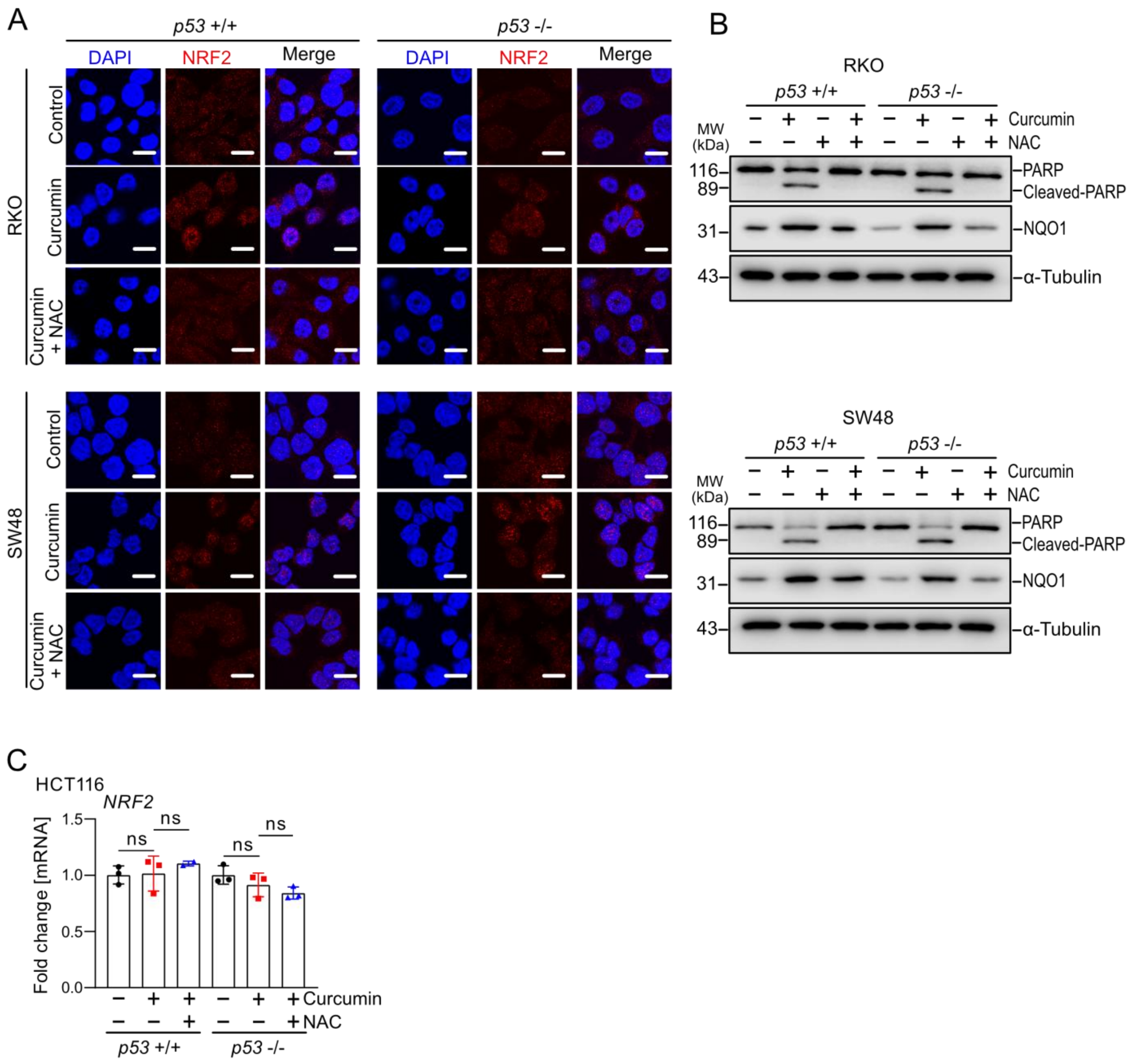

Figure S3: Curcumin activates NRF2 via ROS in CRC cells

A. The localization of NRF2 was detected by immunofluorescence after the indicated treatments. Scale bars: 20 µm. B. Western blot analysis of PARP/cleaved-PARP and NQO1 expression after indicated treatments for 48 hours in RKO (upper panel) and SW48 (lower panel) cells. C. qPCR analysis of the mRNA levels of *NRF2* in HCT116 cells after exposure to curcumin with or without NAC for 48 hours. In panel (C) the mean ± SD (n=3) is provided. ns = not significant.

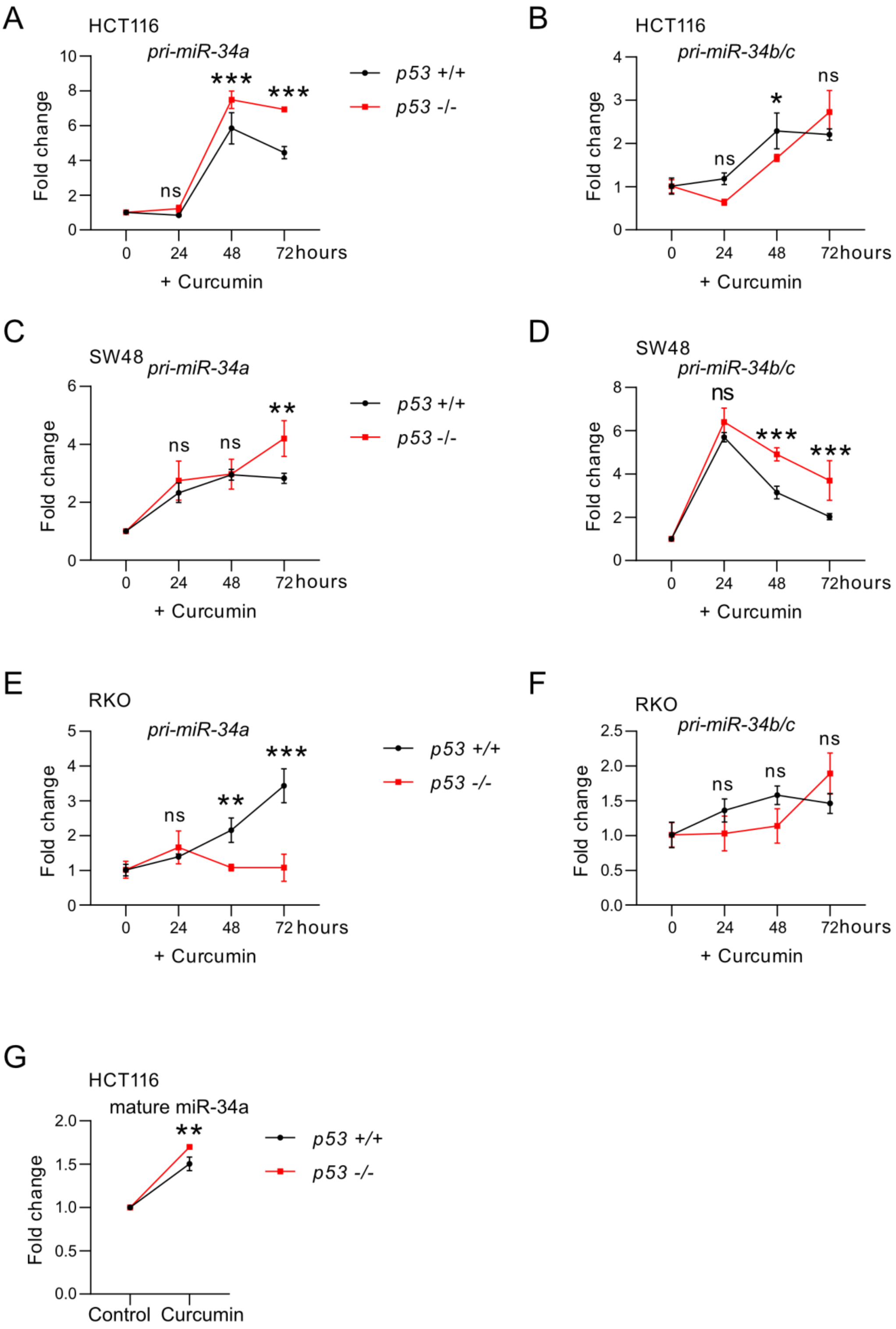

Figure S4: Curcumin up-regulates *miR-34a* and *miR-34b/c* independent of p53
A-F: The expression of *pri-miR-34a* and *pri-miR-34b/c* after treatment with curcumin for indicated periods in indicated *p53*-proficient cells (black line) and *p53*-deficient cells (red line) by qPCR. G. qPCR analysis of mature miR-34a expression in HCT116 cells after treatment with 15 μM curcumin for 48 hours. In panels (A-G) the mean ± SD (n=3) is provided. * $P < 0.05$, ** $P < 0.01$, *** $P < 0.001$, ns = not significant.

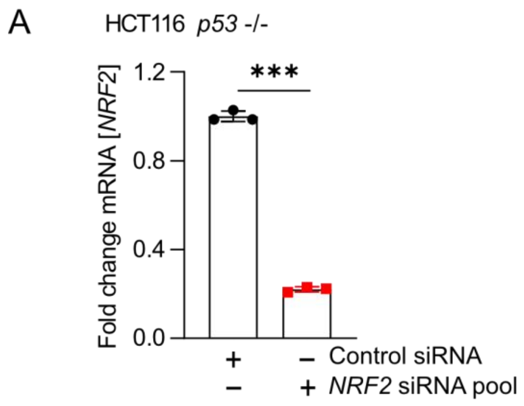

Figure S5: NRF2 directly induces the expression of *miR-34a* and *miR-34b/c*

qPCR (A) and Western blot (B) analysis of NRF2 expression after transfection with control siRNA or *NRF2* siRNA pool in HCT116 p53 -/- cells for 48 h. In panels (A) the mean ± SD (n=3) is provided. *** P< 0.001.

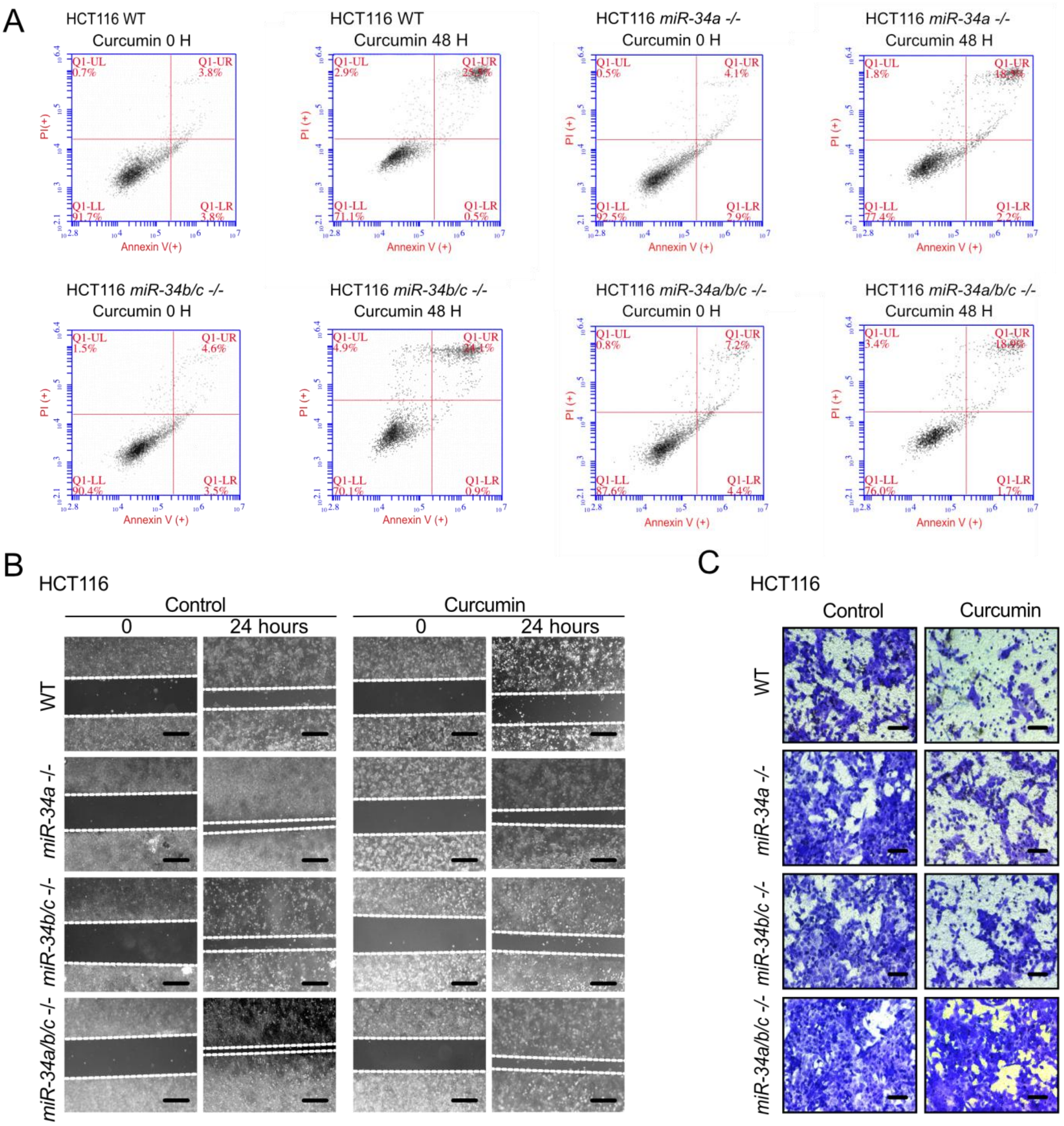

Figure S6: Curcumin promotes apoptosis and inhibits tumor cell migration and invasion via up-regulation of *miR-34* expression

A. Indicated cells were treated with 15 μM curcumin for 48 hours, and apoptotic cells were detected using Annexin V-FITC and PI staining. Cellular migration (B) was detected with wound healing assays and invasion (C) was analyzed by Boyden chamber assays after treatment with curcumin. Scale bars (B): 200 μm. Scale bars (C): 100 μm.

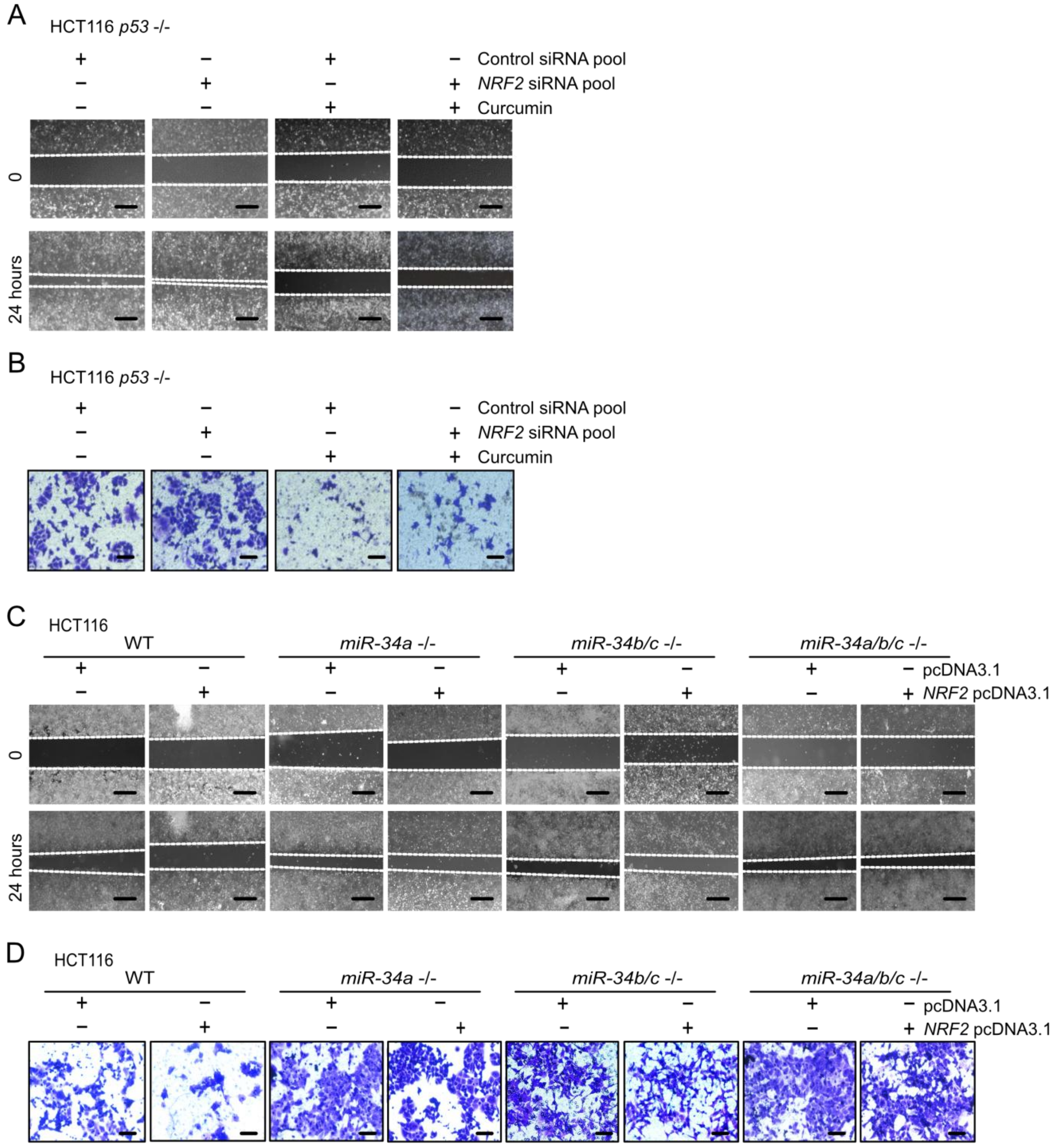

Figure S7: Curcumin repressed the migration and invasion in HCT116 cells via the *NRF2*/*miR-34* axis

A and B. Cell migration (A) analyzed by wound healing assays and invasion (B) analyzed by Boyden chamber assays after exposure to curcumin and NRF2 silencing. (C and D) HCT116 cells were transfected with the pcDNA3.1 and *NRF2* pcDNA3.1 vectors for 48 hours and subjected to a migration wound healing assay (C) for 24 hours and invasion Boyden chambers assays for 48 hours (D). Scale bar (A and C): 200 µm. Scale bar (B and D): 100 µm.

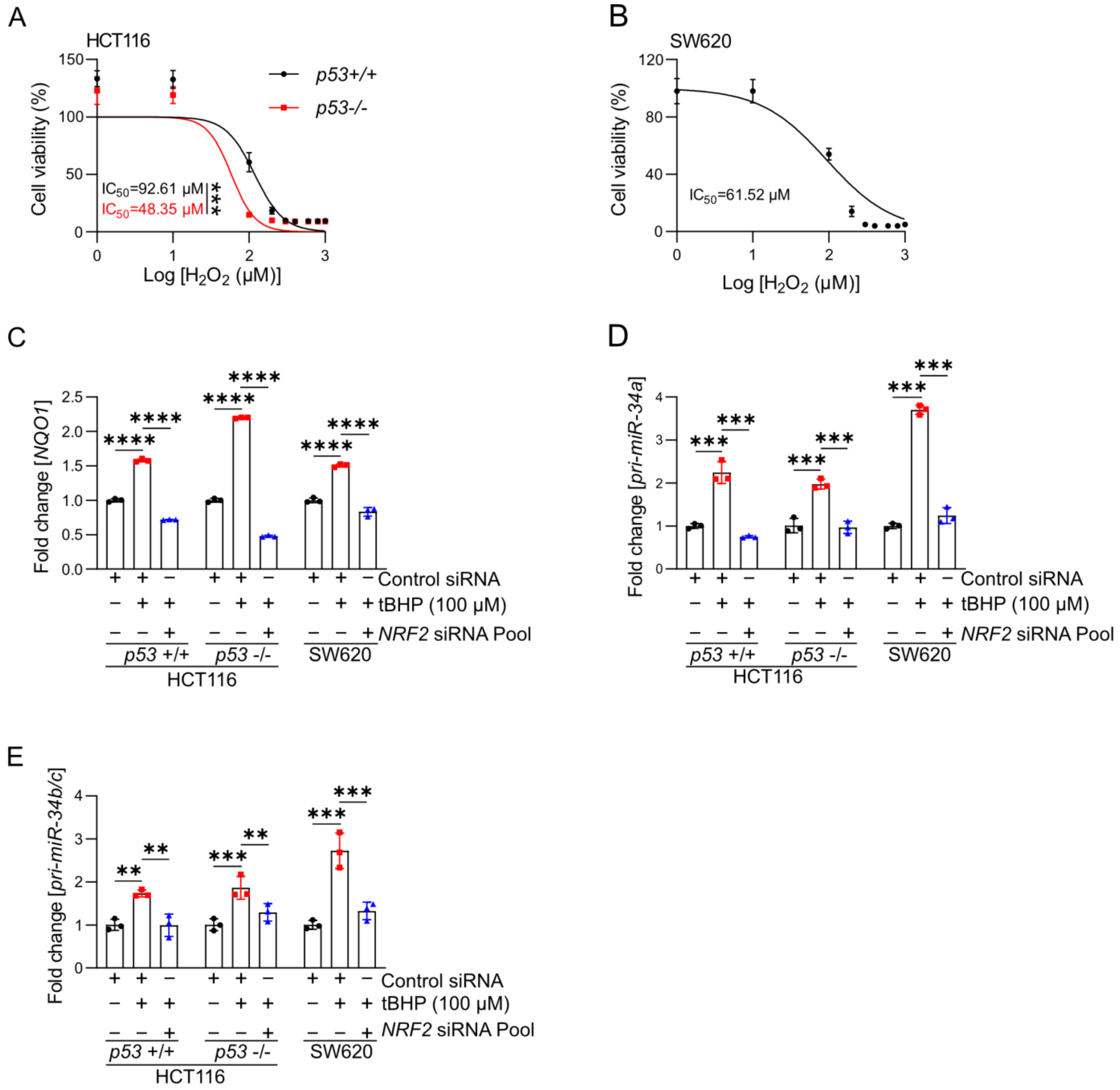

Figure S8: The induction of *miR-34* by tBHP is mediated by NRF2

A and B. HCT116 *p53*-proficient and *p53*-deficient cell lines (A), and SW620 cells (B) were treated with indicated concentrations of H_2O_2 for 48 h. IC_{50} was determined using the MTT method. (C-E) The expression of *NQO1* (C), *pri-miR-34a* (D), and *pri-miR-34b/c* (E) in indicated cell lines after treatment with H_20_2 and transfection with control or *NRF2* siRNA pool.

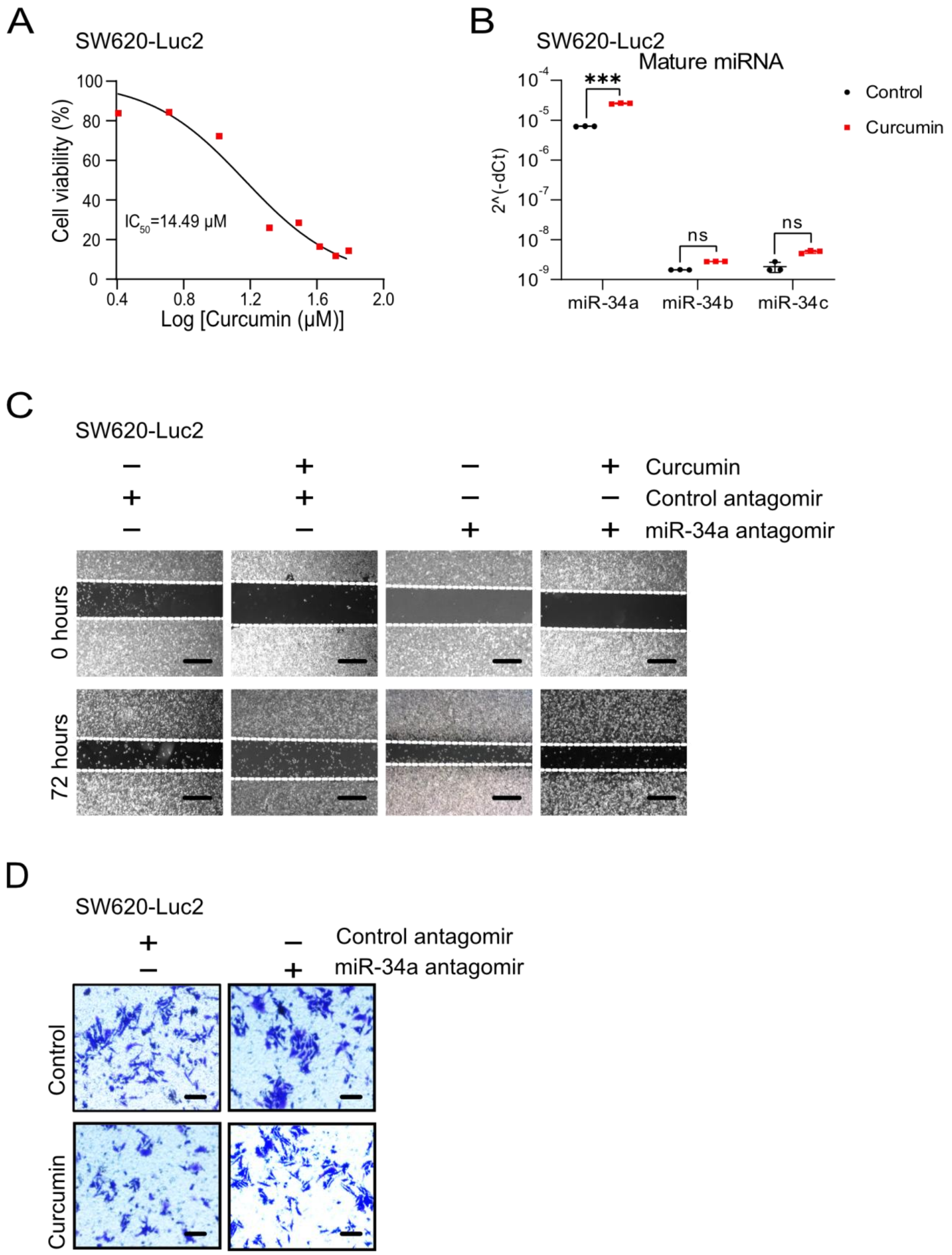

Figure S9: Curcumin inhibits lung-metastases formation via inducing miR-34a
A. SW620-Luc2 cells were treated with indicated concentrations of curcumin for 48 h. IC_{50} was determined using the MTT method. B. Expression of mature miR-34a, miR-34b, and miR-34c was analyzed by qPCR after indicated treatments for 48 hours. C. Cells were subjected to a wound healing assay for 72 hours after the indicated treatments/transfections. Scale bar: 200 μm. D. Cells were subjected to a Boyden chamber assay for 48 hours after the indicated treatments/transfections. Scale bar: 100 μm.

Original Western blots (Uncropped membranes)

Original blots:

Uncropped membranes for Figure 1A

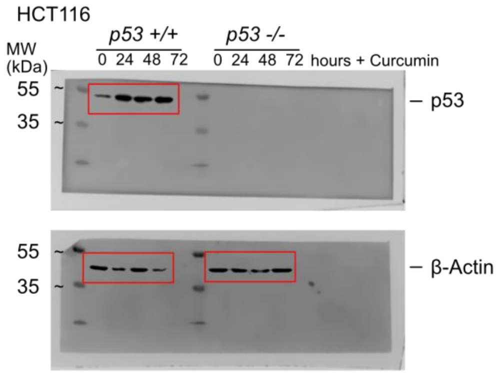

Uncropped membranes for Figure 2C

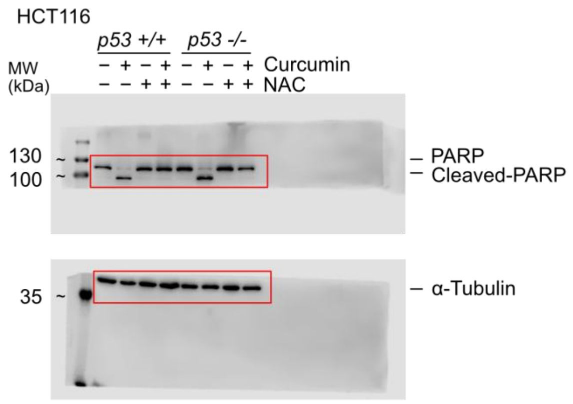

Uncropped membranes for Figure 2E

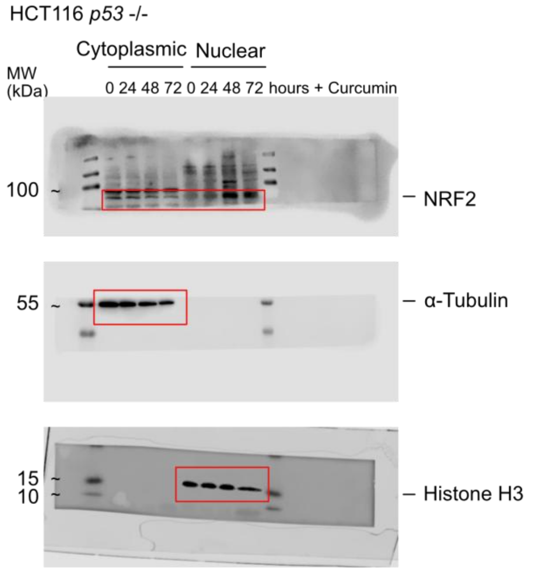

Uncropped membranes for Figure 1G

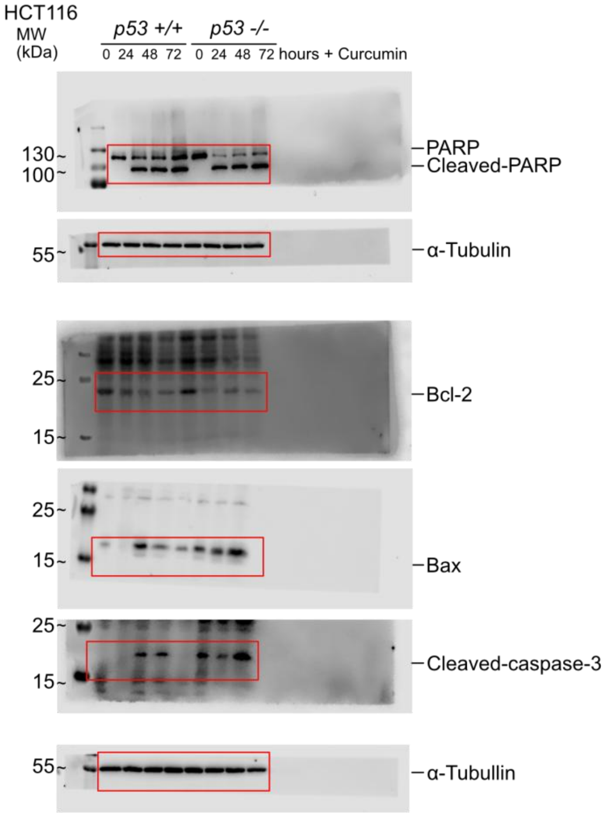

Uncropped membranes for Figure 2G

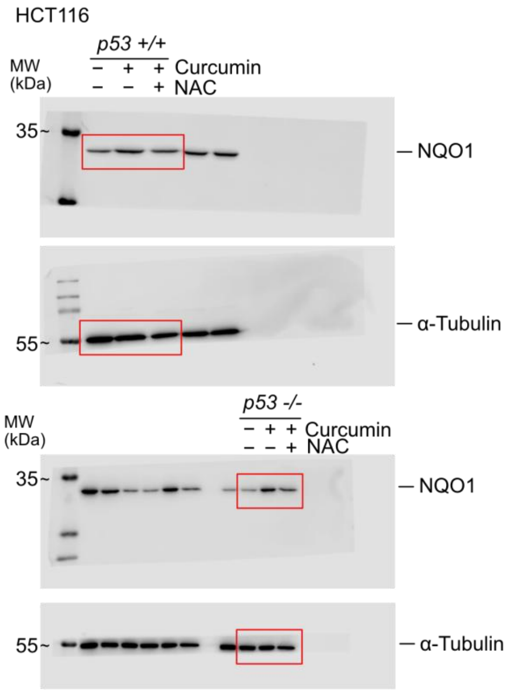

Original blots:

Uncropped membranes for Figure 4C

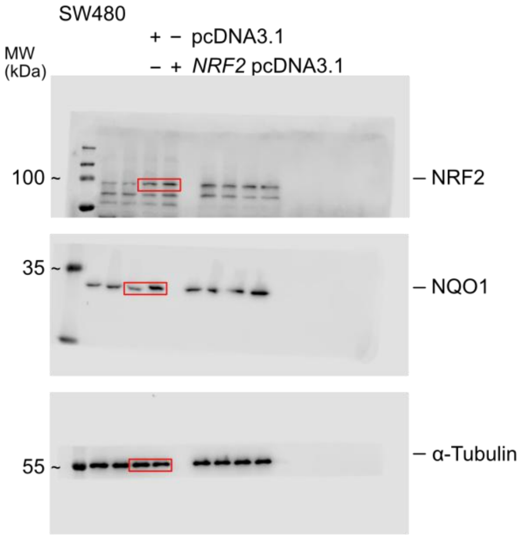

Uncropped membranes for Figure 5C

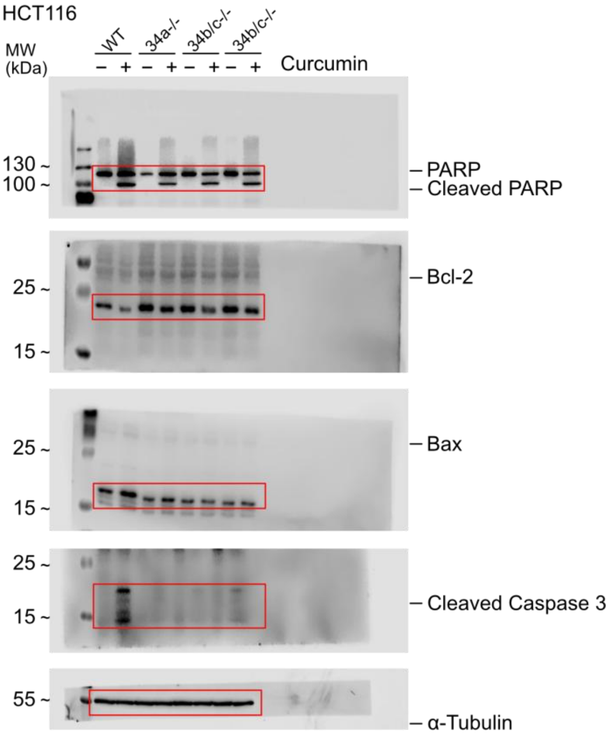

Original blots:

Uncropped membranes for Figure S1G

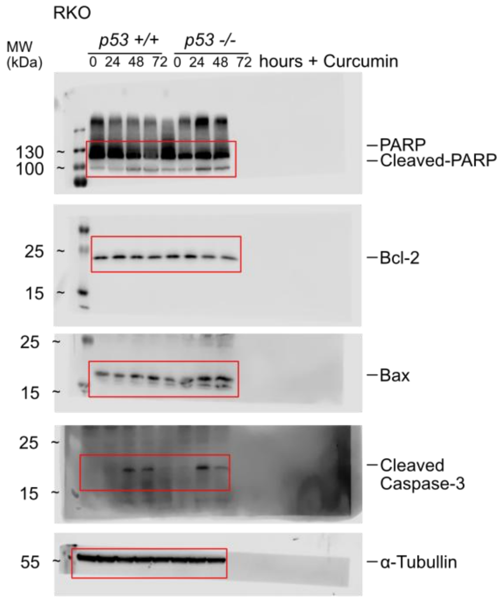

Uncropped membranes for Figure S1H

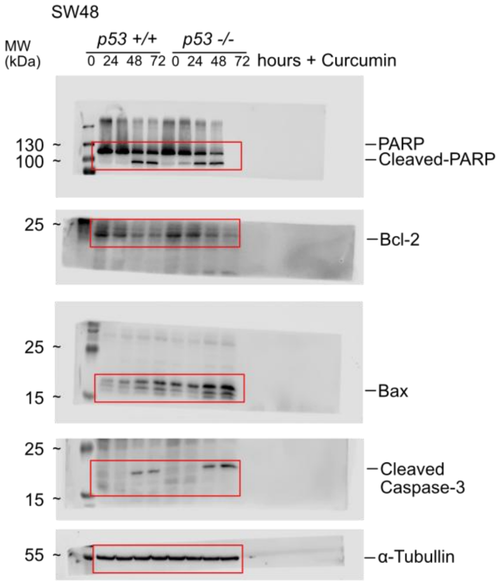

Uncropped membranes for Figure S1F

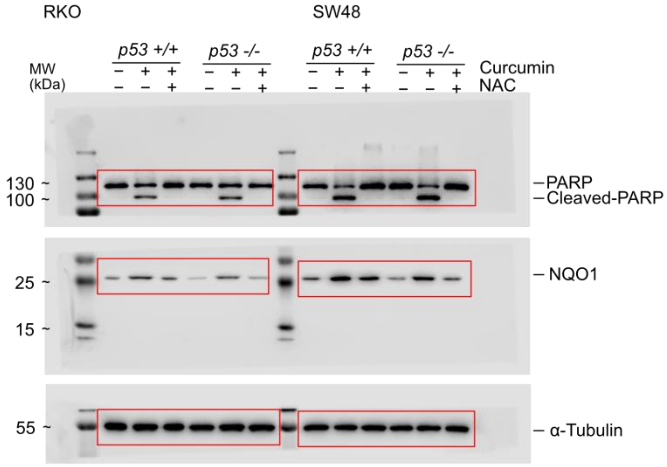

Uncropped membranes for Figure S4

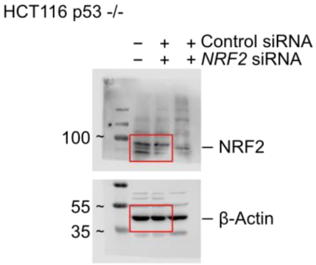

Supplemental Table S1. **Oligonucleotides used for qPCR**

gene	forward (5' – 3')	reverse (5' – 3')
GAPDH	TGTTGCCATCAATGACCCCTT	CTCCACGACGTACTCAGCG
ACTIN	TGACATTAAGGAGAAGCTGTGCTAC	GAGTTGAAGGTAGTTTCGTGGATG
NQO1	TGGTCCCGTTTTGGCTATTCT	GAGACATGCTCCGTGGAGAC
NRF2	GCAAATGAGGTTTCTTCGGC	GGTCTTCTGTGGAGAGGATG
Pri-miR34a	CGTCACCTCTTAGGCTTGGA	CATTGGTGTCGTTGTGCTCT
Pri-miR34bc	GCTCGGTTTGTAGGCAGTGC	GATGGCAGTGGAGTTAGTGA
CDH1	CCCGGGACAACGTTTATTAC	GCTGGCTCAAGTCAAAGTCC
VIM	TACAGGAAGCTGCTGGAAGG	ACCAGAGGGAGTGAATCCAG
SLUG	GGGGAGAAGCCTTTTTCTTG	TCCTCATGTTTGTGCAGGAG
ZEB1	TCAAAAGGAAGTCAATGGACAA	GTGCAGGAGGGACCTCTTTA
SNAIL	GCACATCCGAAGCCACAC	GGAGAAGGTCCGAGCACAC
mature miR-34a	MS00003318	
mature miR-34b	MS00031780	
mature miR-34c	MS00003332	

Supplemental Table S2. Oligonucleotides used for qChIP

gene	forward (5' – 3')	reverse (5' – 3')
MiR-34a (A)	GGACTCCCGCAAAATCTCCA	CACGAGCAGGAAGGAGGAC
MiR-34a (B)	TTACCCCTGGGACCGAGAGA	AGAATCTGTTGCGATGAAATCACT
MiR-34a (C)	TGTCTCAGAACGAGACAGTGG	CCGACTTCGTCCTCTTAGTGA
MiR-34b/c (D)	TGTTGTCTCCAATTGTCTCCA	AGATCGTGCCACTGCACTC
NQO1	ATTCGTCTCCACGGAGCAT	CATGCCCTTTTAGCCTTGG

Supplemental Table S3. List of antibodies

Primary antibodies

epitope	catalog no.	company	use	dilution	source
PARP	# 9532	Cell Signaling Technology	WB	1:1000	rabbit
NRF2	# ab92946	Abcam	WB	1:1000	rabbit
α-Tubulin	# T-9026	Sigma-Aldrich	WB	1:1000	mouse
β-Actin	# 4967	Cell Signaling Technology	WB	1:1000	rabbit
H3	# 9715	Cell Signaling Technology	WB	1:1000	rabbit
NQO1	# sc-32793	Santa Cruz	WB	1:1000	mouse
NRF2	# 12721	Cell Signaling Technology	ChIP<br>IF	1:200<br>1:400	rabbit
P53	# sc-126	Santa Cruz	WB	1:1000	mouse
Cleaved Caspase-3	# 9661	Cell Signaling Technology	WB	1:500	rabbit
Bcl-2	# 3498	Cell Signaling Technology	WB	1:1000	rabbit
Bax	# sc-7480	Santa Cruz	WB	1:1000	mouse
Rabbit IgG		Sigma-Aldrich	ChIP		rabbit

Secondary antibodies or conjugates

name	catalog no.	company	use	dilution	source
anti-mouse HRP	# W4021	Promega	WB	1:10.000	goat
anti-rabbit HRP	# A0545	Sigma-Aldrich	WB	1:10.000	goat
Cy3	ab6939	Abcam	IF	1:2000	goat

Supplementary Methods

Assessment of cell proliferation by real-time impedance measurement

Cell proliferation was assessed by real-time monitoring of electrical impedance (xCELLigence RTCA DP, Roche). Cells were seeded at a density of 3×10^3 cells/well into 100 µl of media in an E-Plate®, which has cross-interdigitated micro-electrodes integrated at the bottom of 96-well tissue culture plate. Curcumin was added to the E-Plates 24 hours after seeding and monitored for additional 116 hours. The cell impedance, referred to as "Cell Index", was automatically calculated on the xCELLigence System and used to generate growth curves. Cell numbers were microscopically determined in a Neubauer chamber at the final time-point.

Cell cycle analysis by flow cytometry

HCT116 cell lines were seeded into 6-well plates at a density of $2x10^5$ cells/ml. 15 µM curcumin was added to CRC cells at various time points. The supernatant was transferred into 15 ml tubes and the plates were incubated once with HBSS. Cells were harvested by adding trypsin and neutralized with 1 ml of fresh medium. The cell suspension was transferred to a 15 ml tube, centrifuged at 1200 rpm for 4 minutes, and the supernatant was discarded. Cells were washed with HBSS once and then 1 ml of 70% alcohol was added dropwise to the pellet. Then 7 ml HBSS was added. After centrifugation 400 µl PI staining solution was added to the dissolved pellet for 4 hours. Cells were analyzed by flow cytometry using a CFlow6 (device (Accuri, Ann Arbor, MI).

Detection of apoptosis by flow cytometry

HCT116 cells were seeded into 6 well plates at a cell density of $2x10^5$ cells per well. After 24 hours cells were treated with curcumin (15 µM) and collected after 24, 48, and 72 hours. Cells were washed with HBSS and resuspended in 1 X binding buffer. FITC Annexin V and PI was added and incubated for 15 minutes at room temperature in the dark. After addition of 1x binding buffer, cells were analyzed by flow cytometry using a CFlow6 (device (Accuri, Ann Arbor, MI).

Western blot analysis

Cells were washed by HBSS and lysed in RIPA lysis buffer (50 mM Tris/HCl, pH 8.0, 250 mM NaCl, 1% NP40, 0.5% [w/v] sodium deoxycholate, 0.1% SDS, complete mini protease inhibitors [Roche, Basel, Switzerland] and PhosSTOP Phosphatase Inhibitor Cocktail Tablets [Roche]). Lysates were sonicated for 5 seconds and centrifuged at 13000 rpm for 20 minutes at 4℃. Supernatants containing proteins were transferred to new tubes and quantified by Pierce™ BCA Protein Assay Kit (Termo Fisher Scientifc). 40 µg of protein from each sample was loaded and separated on 10% SDS-acrylamide gels. Gel electrophoresis and transfer to Immobilon PVDF membranes (Millipore, Burlington, MA, USA) were performed according to manufacturer's instructions (BioRad Laboratories, Hercules, CA). Membranes were blocked with non-fat dry milk. For immuno-detection, the membrane was incubated with the primary antibodies listed in Table S3. The signal from the HRP-conjugated secondary antibody was generated by enhanced chemi-luminescence (Millipore) and detected using a LI-COR Odyssey Fc imaging system (LI-COR, Lincoln, NE).

Beta-galactosidase (β-gal) staining

β-gal staining was performed according to manufacturer's instructions using a beta-galactosidase staining kit (Abcam, USA). Cells were seeded in 12 well-plates with a density of $1x10^5$ cells/ml. Cells were treated with curcumin for 48 hours, washed with PBS and fixed with fixative solution for 15 min at room temperature. Fixed cells were washed with PBS two times and subjected to 0.5 ml of the staining solution mix. Plates were incubated overnight at 37°C and imaged by using a microscope (Axiovert 25, Zeiss, Jena, Germany) with Axiovision software (Version 4.8.0.0, Zeiss).

Modified Boyden-chamber assay

For determination of invasion chamber membranes with 8.0 µm pore size (Corning, NY) were coated with 60 µl Matrigel (BD Biosciences, East Rutherford, NJ) at a dilution of 3.3 ng/mL in a medium without serum before the cell suspension in serum-free medium ($5x10^4$ cells) was added to the upper chamber for 4 hours. Curcumin (15 µM) or DMSO was added to the top of the chambers. Then 500 µl of 10% FBS medium was added to the lower chamber. After 48 hours, the invaded cells located on the lower surface of the cell culture inserts were fixed, stained with crystal violet, and counted. Fold changes in invasive cells were calculated by normalizing them to the corresponding control group.
